# Cartography of rhodopsin-like G protein-coupled receptors across vertebrate genomes

**DOI:** 10.1038/s41598-018-33120-8

**Published:** 2019-05-07

**Authors:** Maiju Rinne, Zia-Ur-Rehman Tanoli, Asifullah Khan, Henri Xhaard

**Affiliations:** 1 Drug Research Program, Division of Pharmaceutical Chemistry and Technology, Faculty of Pharmacy, University of Helsinki, P.O. Box 56, FI-00014 University of Helsinki, Helsinki, Finland; 2 Department of Computer and Information Sciences, Pakistan Institute of Engineering and Applied Sciences (PIEAS), P.O. 45650, Nilore, Islamabad Pakistan

**Keywords:** G protein-coupled receptors, Data mining

## Abstract

We conduct a cartography of rhodopsin-like non-olfactory G protein-coupled receptors in the Ensembl database. The most recent genomic data (releases 90–92, 90 vertebrate genomes) are analyzed through the online interface and receptors mapped on phylogenetic guide trees that were constructed based on a set of ~14.000 amino acid sequences. This snapshot of genomic data suggest vertebrate genomes to harbour 142 clades of GPCRs without human orthologues. Among those, 69 have not to our knowledge been mentioned or studied previously in the literature, of which 28 are distant from existing receptors and likely new orphans. These newly identified receptors are candidates for more focused evolutionary studies such as chromosomal mapping as well for in-depth pharmacological characterization. Interestingly, we also show that 37 of the 72 human orphan (or recently deorphanized) receptors included in this study cluster into nineteen closely related groups, which implies that there are less ligands to be identified than previously anticipated. Altogether, this work has significant implications when discussing nomenclature issues for GPCRs.

## Introduction

G protein-coupled receptors (GPCRs) are signalling proteins activated by for example neurotransmitters and neuromodulators and as such are involved in many physiological processes. Their location as a cellular gateway makes them key targets for drug discovery and chemical biology^1–3^, and 30–50% of drugs on the market have been reported to target GPCRs directly or indirectly^4^.

GPCRs are well known for sharing a three-dimensional architecture characterized by seven transmembrane α-helical segments^5,6^ connected by loops. In the rhodopsin family, a well conserved disulphide bridge often connects the second extracellular loop and the third transmembrane segment. This architecture is reflected at the sequence level by conserved sets of amino acids that serve structurally or as determinants of signal transduction, e.g. to name a few the E/DRY, CWxP, NPxxY motifs in the transmembrane segments 3, 6 and 7 of the rhodopsin family^7,8^. Additional family-specific motifs have been identified for examples in connecting loops^9^. Within each transmembrane segment, the most conserved amino acid will be referred to here as pivots (pivot amino acids are not always fully conserved: N1.50, 98% conservation; D2.50, 90%; R3.50, 95%; W4.50, 97%; P5.50, 78%; P6.50, 99%; P7.50, 88% see^7^). These amino acids are used as a basis of the widely used Ballesteros-Weinstein numbering^10^, where equivalent positions are reported by transmembrane segment number followed by relative distance to pivot, itself assigned the index 50.

The International Union of Basic and Clinical Pharmacology (IUPHAR) is responsible for the international classification of GPCRs and issues regular recommendations. GPCRs have been divided into five main families based on phylogenetic analyses^11–13^. The largest, *Rhodopsin*, in human ~700 members, subdivides into four branches (α, β, γ and δ) and 13 sub-branches^11^ and has originated through local duplications about 1400–1100 million years ago^14^. Families are further divided into subtypes^15^ that arouse, for many, 350–500 million years ago from two rounds of whole genome duplication (2R)^16–22^. In ray-finned fish (not in lobe-finned or jawless fishes), a third specific genome duplication took place about ~250 million years ago, leading to fish-specific duplicates^23–25^. In tunicates, GPCRs originated before the 2R and after the separation of invertebrates from chordates^24–26^. In lamprey, it remains unclear whether the cyclostome-gnathostome split at the origin of jawed vertebrates happened before or after the second round of genome duplication^27–29^.

As consequence of the origin of vertebrate GPCRs through the 2R/3R, in an ideal scenario, for each ancestral receptor we expect four/eight paralogues (that can be referred to as “ohnologues”^16^, many ohnologues are simply equivalent to subtypes). Nonetheless, local duplications and deletions (pseudogenes)^30^ may make the current day picture complex and, consequently, the expect four/eight ohnologues are most often not seen^18,30^. Furthermore, ligand binding preferences do not necessarily indicates the closest possible evolutionary relationships; some receptors have acquired the same ligand specificity several times, for example α_1_-, α_2_-, and β- adrenoceptors that are parts of the amine family^31,32^, or e.g. the cannabinoid receptors CB_1_ and CB_2_, and the recently deorphanized GPR_55_ that are activated by the same endogenous ligand 2-Arachidonoylglyserol^33^.

GPCRs have been identified and characterized in the 70’s and cloned in the 80’s, e.g. the well-studied rhodopsin^34^ and β_2_-adrenergic receptor^35^. The apparition of genomics in the 2000’s has brought an explosion in the amount of discovered GPCRs, yet GPCRs are in most cases named according to their human orthologues^26,36–38^. This leads to complex nomenclature issues especially when new clades without human orthologues are identified^39–41^. In addition, the repertoires in non-human vertebrate species are much less explored, and new subtypes or orphan receptors are often not annotated at all. The only way to date to tackle the issue of GPCRs in non-vertebrate species is the tedious manual annotation based on phylogenetic information.

Here, we conducted this manual annotation based on the online Ensembl database. The transcripts predicted in Ensembl are based on automated alignments matched to curated homologues sequences or ESTs. Ensembl gene trees are based on a consensus of five tree reconstruction methods: a maximum likelihood based on two types of distances and a neighbor-joining tree based on three types of distances. This annotation is a first step towards an experimental classification that would include cloning, pharmacological characterization, as well as a chromosomal analysis.

## Results and Discussion

### Overview

All data used for this study are available online and can be easily accessed using the codes provided (see the Experimental section).

This study was conducted based on the Ensembl genomic data^42,43^ twice with a five-year interval: 2012–2013, Ensembl release 67 (referred to as Ensembl.R67); and 2017–2018, Ensembl.R90-Ensembl.R.92. The text is organized according to the Ensembl.R91 trees (December 2017, available as an archive, see Experimental section). Some of the groupings may slightly differ in future releases. Ensembl.R92 (April.2018) became available during the final preparation of this manuscript and was used to solve a few ambiguities, as mentioned in text. The proposed new gene symbols were submitted to HUGO (Human Genome Nomenclature Committee) for initial consideration; for this purpose the suggested genes symbols were reevaluated against the Ensembl.R.94 (December 2018) and Ensembl.R.95 (February 2019) releases. They will be submitted to the other relevant committees on publication (MGNC, Mouse Genomic Nomenclature Committee; RGNC, Rat Genome and Nomenclature Committee, ZNC, Zebrafish Nomenclature Committee; XGC, Xenopus Gene Nomenclature Committee; CGNC, Chicken Gene Nomenclature Consortium). Therefore all gene symbols presented in this manuscript should be regraded as tentative until approved by the relevant committees.

Altogether, in Ensembl.R92 we identified 142 clusters of genes corresponding to non-olfactory rhodopsin-like GPCRs without human orthologues (Tables 1, 2), of which 69 have not been to our best knowledge previously described. Twenty-eight are distant from any group of receptors and likely orphan, and the others probable subtypes of existing receptors (Table 2). Not surprisingly, most of the new receptors are present in ray-finned fishes and only a few in placental mammals (Table 1). Nonetheless, most (23) of the new orphan receptors are found in two or more species clades (considering separately ray-finned and lobe-finned fishes, amphibians, birds, reptiles, monotreme, marsupial and placental mammalians). In addition, families that are evolutionary more recent and faster evolving such as purine (PUR) and chemokine (CHEM) contains a larger number of previously unidentified receptors (Table 2). We furthermore identify groups of ambiguous branching that need to wait for more and/or increased quality sequence data to be characterized (not annotated, see text below).

**Table 1:** Species-specific counts of non-olfactory GPCRs clades without human orthologues in Ensembl.R91.

Representative species	Lamprey	Fishes (*Actinopterygii*)		Fishes (*Sarcopterygii*)	Amphibian	Reptiles	Birds	Mammals		
	*Petromyzon marinus*	Cypriniformes (*Danio rerio*); Characiformes (*Astyanax mexicanus*)	*Lepisosteus oculatus* + *Takifugu rubripes* and 7 others	*Latimeria chalumnae*	*Xenopus tropicalis*	*Anolis carolinensis; Pelodiscus sinensis*	*Gallus gallus* +4 others	Monotreme	Marsupial	Placental
Whole genome duplication	1R/2R?	3R	3R, except *Lepisosteus oculatus* 2R	2R	2R	2R	2R	2R	2R	2R
Pictogram										
Number of genomes	1	2	9	1	1	2	5	1	3	65
Paralogous GPCR clades identified	22	118	134	95	69	74	52	16	19	11

**Table 2:** Family-specific counts of non-olfactory GPCRs clades without human orthologues in Ensembl.R91.

	AMIN	MECA	PTGER	MLT	OPN	PEP	CHEM	SOG & MCH	LGR	MRG	PURIN	Others	Total
Subtypes	13	5	1	20	3	24	12	13	2	3	20	7	123
Orphans	2	1	1	0	0	1	5	2	0	0	10	2	24
Total orphan + subtypes	15	7	2	20	3	25	17	15	2	3	30	9	147
Including, novel	6	3	1	1	0	9	7	3	0	0	27	8	66

There are few aspects to keep in mind regarding manual annotations. First, we do not have enough manuscript space to detail the annotation of each of the 142 new receptor clades. Two examples are given as Supporting information. The annotation process is in our opinion straightforward enough to be reproducible by the reader using the online data. Secondly, this is not a full characterization of the novel receptors, which would require pharmacological studies and chromosomal organization studies. Thirdly, sometimes the branching alone do not allow a distinction between subtypes or new orphan receptor clades. Fourthly, small discrepancies may exist between the guide trees and the text of this article, essentially because the guide trees are older (R.67).  We encourage the readers to look at the most recent data and build an informed opinion.

### Amine and trace amine receptors (AMIN)

**Figure 1:**
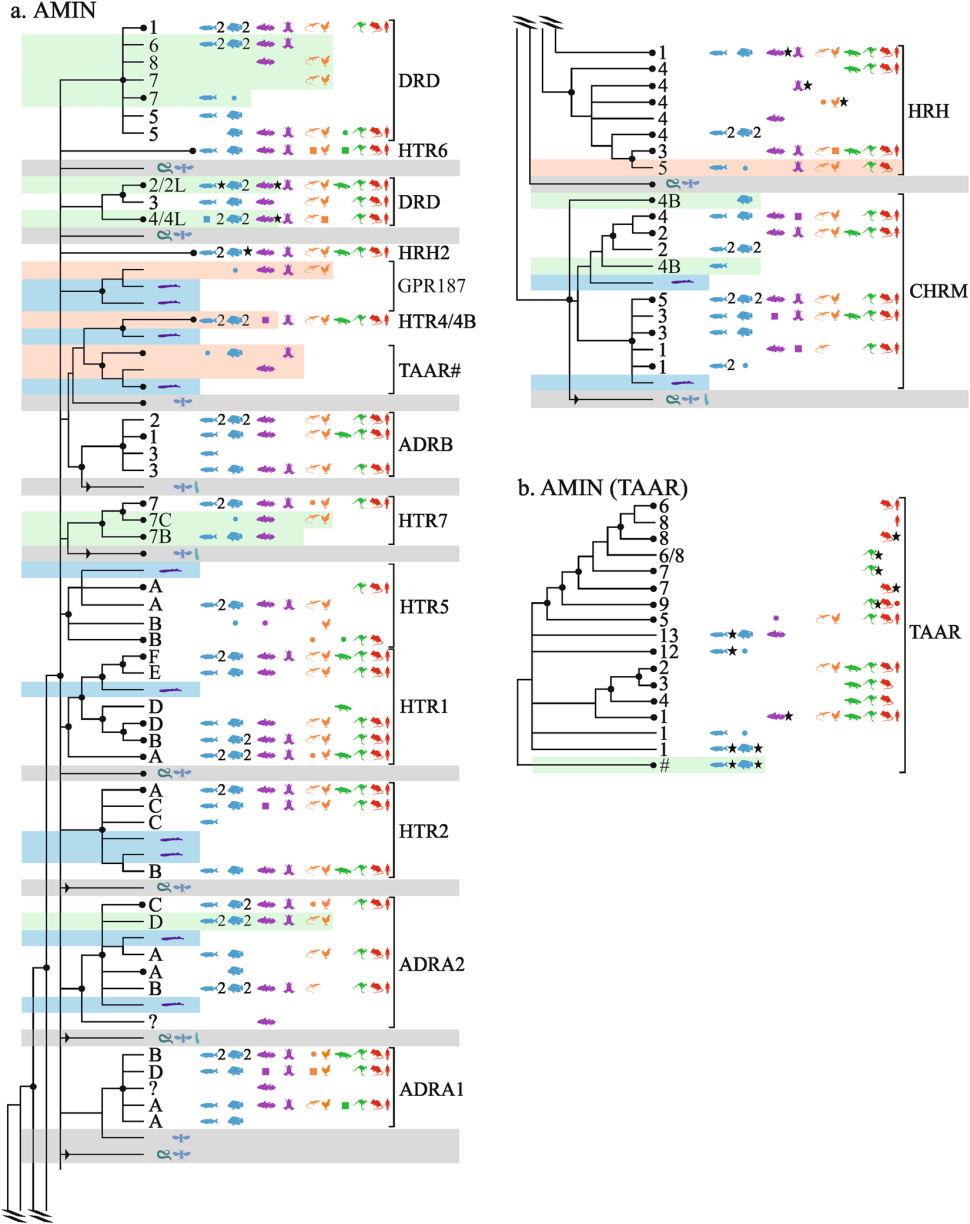
Overview of vertebrate GPCR clades mapped onto neighbor-joining trees of the α-branch (1/2). AMIN (**a**,**b**). Some branches are collapsed together. Species symbol, see Table 1. Squares, genes mistakenly removed during the curation process; circles, sequences of a representative species added in Ensembl.R90-R91 releases. Internal duplications (stars), presence of 3R duplicates (number “2”). Isolated non-assigned sequences (“?”) or genes in Ensembl.R67 that are not found in further releases (“Δ”). Bootstrap values above 65% (black circles). Highlights: new receptors that have (green) or have not (light red) been to our knowledge previously reported; presence of putative ancestral sequences from lamprey (light blue) or early vertebrates or invertebrates (light grey). Some branches have been collated together.

In the AMIN family (Fig. 1a,b) we suggest one new orphan receptor and four new subtypes that to our best knowledge have not been previously reported. This family was among the first discovered and its evolution has been comprehensively studied (see e.g.^44^), in particular, for dopamine receptors^40,45^.

#### Gene tree ADRA2A,2B, 2C; ADRB1,2,3; DRD1,5; DRD2,3,4; HTR2A,2B,2C; HTR6; HRH2

In the dopamine D_2,3,4_ receptor family, there are two new fish-specific clades found in coelacanth and ray-finned fishes: see ENSDARP00000127653, named here D_2L_ (gene: DRD2L)(not to be confused with the long splice variant of D_2_); and see ENSAMXP00000016690, named D_4L_. Previous work characterized the expression in zebrafish of a single D_3_ and three D_2_ receptors^46^, whereas this study suggest seven zebrafish receptors in the D_2,3,4_ family: D_2a_, D_2b_, D_2L_, D_3_, D_4a_, D_4b_, and D_4L_. A previously cloned and pharmacologically characterized α_2_-adrenoceptor D subtype (ADRA2D) subtype was also found^9,47^. Near the histamine receptor H_2_ (HRH2), rooted by two lampreys, a gene cluster from gar/coelacanth/amphibian/sauropsids(7), see ENSPMAP00000006842, was named GPR_187_.

#### ADRA1A,1B,1C; HTR1A,1B,1C,1D,1E; HTR5,7

We identified at least two unannotated 5-hydroxytryptamine subtypes: 5-HT_7B_ (HTR7B) found in gar/fishes(10)/coelacanth, see ENSLACP00000008875, that has likely been cloned in zebrafish^48^; and a set of genes in gar/coelacanth/sauropsids(7), see ENSLACP00000011078, that we name 5-HT_7C_ (HTR7C). A cluster of five genes from gar/coelacanth/birds(3), see ENSLOCP00000003684, may be orthologues of the mammalian 5-HT_5B_. The 5-HT_5B_ is pseudogenic in humans, but well characterized in mice^49^.

#### HRH1,3,4; CHRM1,2,3,4,5

In the histamine H_1,3,4_ subtree, a set of genes from gar/fishes(2)/amphibian/sauropsids(7)/mammals(4, 2 marsupials), see ENSLOCP00000006664, was named H_5_ (HRH5). Near the muscarinic cholinergic receptors 4 and 2, a group of ray-finned fish genes (8), see ENSDARP00000128513, was named CHRM_4B_; these muscarinic receptors have been cloned from zebrafish^50^.

#### HTR4; TAAR

Equally distant from 5-HT_4_ and trace amine (TAAR) receptors, a monophyletic group containing fishes(9)/coelacanth/gar/amphibian, see ENSLACP00000014144, was left unnanotated. Near the 5-HT_4_ subtree, a set containing gar/fishes(10)/coelacanth genes, see ENSLACP00000004821, was named 5-HT_4B_ (gene HTR4B; not orthologous to human 5-HT_4_ since this later has spotted gar). The TAAR have been studied in mouse, rat, human and chimpanzee^51^ and fishes, where their repertoire is substantially larger than in human^52,53^. A set of ray-finned fish genes, see ENSLOCP00000022119, may belong to a previously unannotated subtype.

### Melanocortin/EDG/Cannabinoid/Adenosine (MECA) receptors

**Figure 2:**
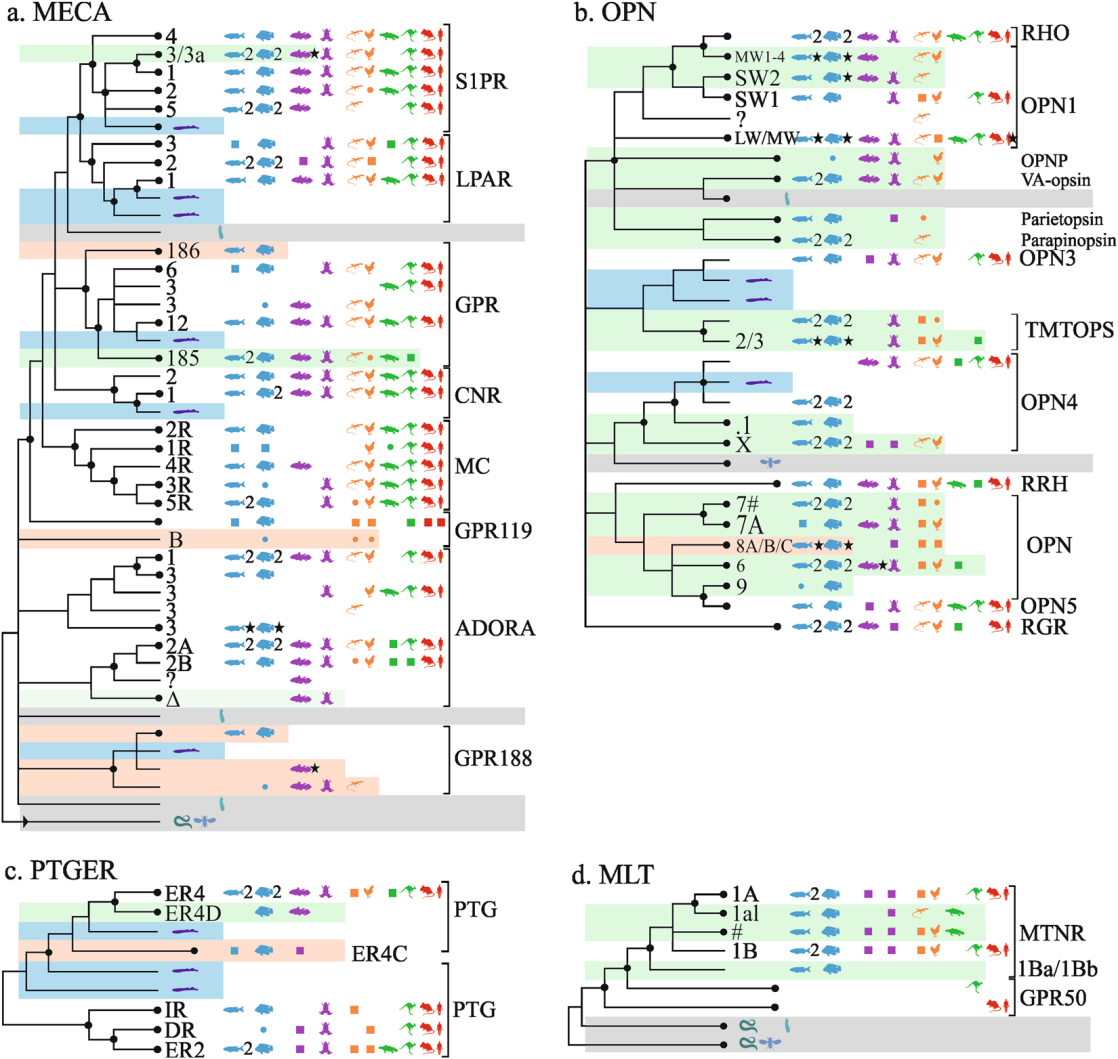
Overview of vertebrate GPCR clades mapped onto neighbor-joining trees of the α-branch (2/2). MECA (**a**); OPN (**b**); PTGER (**c**); MLT (**d**). Caption otherwise similar to Fig. 1.

In the MECA family (Fig. 2a) we identified one new orphans, a new subtype, and a fish-specific receptor clade.

#### ADORA1,2,3

Two sets of genes are likely subtypes of a new orphan receptor, GPR_188_, rooted by lamprey and ascidian sequences, see ENSCINP00000025465. The first set contains gar/coelacanth(duplicated)/fishes(9), see ENSLOCP00000022091; and the second includes fishes(2)/coelacanth/amphibian/reptiles(2), see ENSGMOP00000016757. The duplicated coelacanth sequences open the possibility of a third subtype, but not conclusively.

#### MC1,2,3,4,5R; GPR119

The orphan receptor GPR_119_ has a potential subtype named GPR_119B_, found in gar/sauropsids(5), see ENSLOCP00000002170.

#### S1PR1,2,3,4; LPAR1,2,3; GPR3,6,12; CNR1,2

A cluster of ray-finned fishes (9) genes suggest a new receptor close to GPR_3_, see ENSAMXP00000025994. Nearby, another set has genes of gar/fishes(9)/coelacanth/amphibian/sauropsids(3)/marsupial(1)/monotreme(1), see ENSLOCP00000021577, and corresponds to GPR_185_; this receptor has been cloned in *Xenopus laevis* (named also GPR_x_^54^). Two novel sphingosine-1-phosphate receptor subtypes have been previously reported in zebrafish^55^, which should correspond here to a set of fishes(17)/coelacanth/reptile(1) and to S1PR_3a_ in fishes(10)/coelacanth.

### Opsin (OPN) receptors

In the OPN family (Fig. 2b), one new fish-specific opsin was identified. OPN has been extensively studied, in particular cone visual pigments^56^, UV-sensitive photoreceptors^57^, and melanopsins^58^. Zebrafish has 10 classical visual photo pigments and 32 non-visual opsins^57,59^.

#### OPN1MW/LW/SW; OPN3/4/5; RHO; RRH

Near OPN_8a_, receptors from a set found in gar/fishes(7), see ENSLOCP00000020544, were named OPN_8b_. They come in addition to the OPN_4–9_ in zebrafish^59^.

### Prostaglandin (PTGER) receptors

In the PTGER family, one new subtype was identified (Fig. 2c).

#### PTGER1,2,3,4; PTGDR; PTGIR; PTGFR; TBXA2R

Near PTGER_4_, a group of fishes(7)/coelacanth/spotted gar, see ENSLACP00000020254, is a probable new subtype, PTGER_4D_ that has been previously characterized in zebrafish^60^. In addition, a group of gar/fishes(11)/coelacanth genes, rooted by lamprey, see ENSAMXP00000018684, suggests a new subtype PTGER_4C_.

### Melatonin (MLT) receptors

In the melatonin tree, no new receptors could be identified (Fig. 2d).

#### MLT, GPR50

Ambiguities in this tree were lifted in Ensembl.R92. A complete set containing gar/fishes(10)/coelacanth/amphibian(duplicated)/sauropsids(7)/platypus, see ENSLOCP00000018152, clusters with GPR_50_ (data from R.95).  Another set includes lamprey/gar/fishes(9)/amphibian/sauropsids(2)/platypus, see ENSLOCP00000014362, was named MTNR_1al_. A third set of fishes (4), see ENSDARP00000070419, was named MTNR_1Ba/1Bb_. Three MLT subtypes have been previously cloned in zebrafish^61^.

### Peptide (PEP) receptors

**Figure 3:**
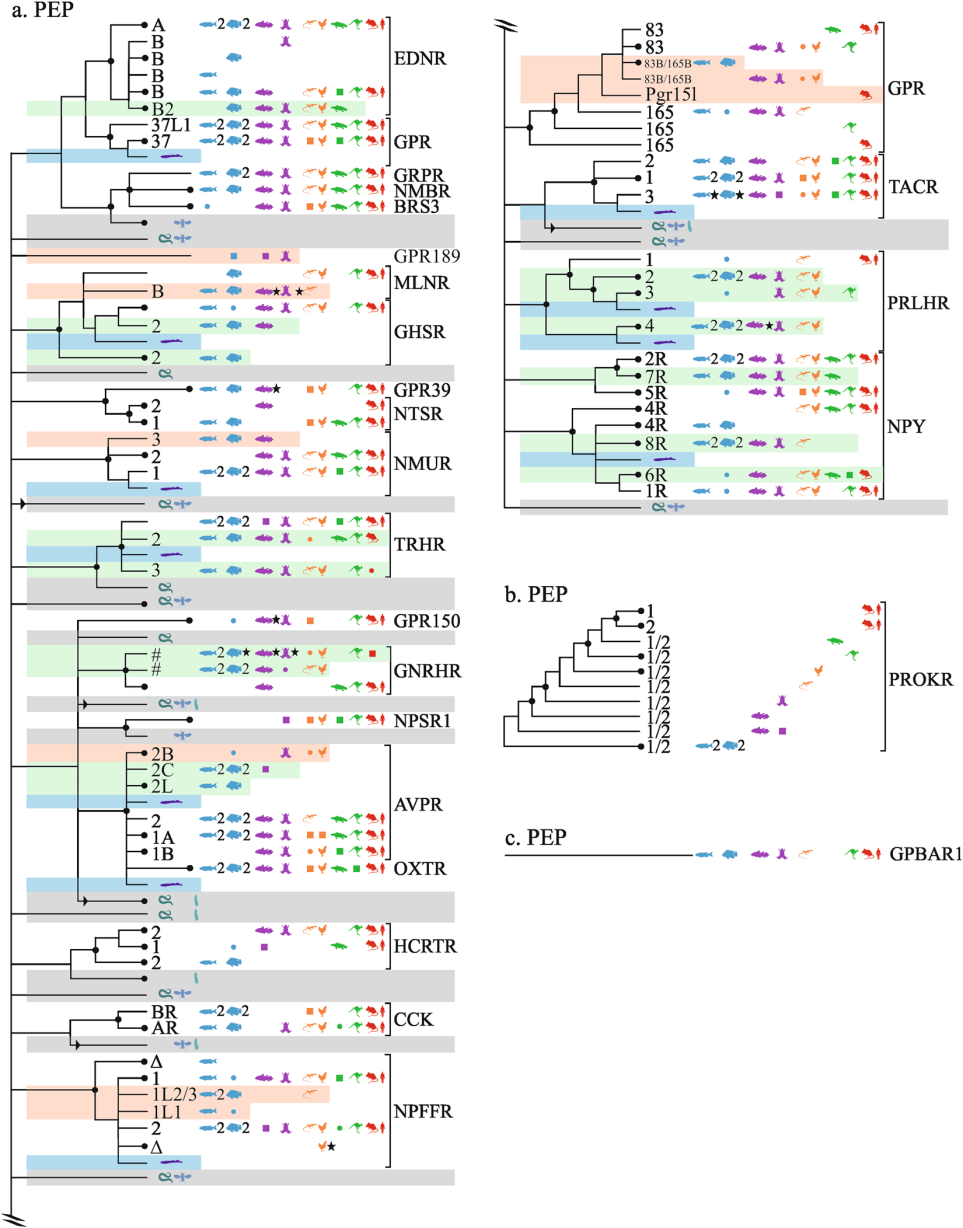
Overview of vertebrate GPCR clades mapped onto neighbor-joining trees of the β-branch. PEP receptors. Caption otherwise similar to Fig. 1.

In the PEP family (Fig. 3a–c), we identified ten vertebrate receptors without human orthologues, one of which is likely a new orphan. PEP is generally conserved, retaining many lamprey sequences at the root of receptor clades.

#### NTSR, GPR39, MLNR, GHRS, NMUR, TRHR

Near GPR_39_ and neurotensin receptors (gene: NTSR), rooted by lamprey, a set of gar/fishes(3)/coelacanth/amphibian genes, see ENSXETP00000007421, suggest a new orphan, GPR_189_.

The motilin receptor (gene: MLNR) has one novel subtype, MLNR_B_ that contains coelacanth/amphibian/reptiles(2), see ENSLACP00000009190. A closely branched set of gar/fishes(9) genes, see ENSLOCP00000011352, may additionally belong to MLNR_B_.

The growth hormone secretagogue receptor (GHSR) tree includes three groups of fishes: one containing gar/zebrafish, see ENSLOCP00000009072, likely orthologous to human GHSR; one containing gar/fishes(10)/coelacanth, see ENSLOCP00000000036, that was named GHSR_2_; and a set of fishes (11, duplicates in cave fish), see ENSAMXP00000003803, also orthologous to human GHSR. Four goldfish receptors (GHS-R1a type 1/type 2, GHS-R2a type 1/type 2) have been previously suggested^62,63^.

The thyrotropin-releasing hormone receptors 1–3 (TRHR) are resolved in Ensembl.R92 as one orthologue and two paralogues of the human TRHR receptor. Four receptors (THR_1a_, THR_1b_, THR_2_, THR_3_) have been previously characterized in medaka fish^64^ and three cloned in frog^65^.

The neuromedin U receptors (NMUR) are divided into NMUR_1_ and NMUR_2_, both with lamprey orthologues (ENSPMAP00000009319, ENSPMAG00000001504). Two sets of fish genes were assigned to NMUR_1_ (based on Ensembl.R92). A set of sequences found in lamprey (ENSPMAP00000007962) and gar/fishes(10)/coelacanth, see ENSLOCP00000004052, was named NMUR_3_.

#### NPFF; QFRPR; HCTR; NPY4,8,6,1; NPY5; NPY7; NPY2R; PRLHR

Two groups of genes are likely ohnologues of NPFFR_1_: a gar/fishes(11) set, see ENSLOCP00000019807, named here NPFFR_1L2/3_; and gar/fishes(3), see ENSLOCP00000018374, named NPFFR_1L1_. Two reptile genes branch with either of these receptors.

Two groups of two novel prolactin releasing hormone receptors (PRLHR) are found: the first group is rooted by lamprey (ENSPMAP00000011235) and comprised of one set comprising gar/fishes(16, duplicated)/coelacanth/amphibian/birds(3), see ENSLOCP00000018414, named here PRLHR_2_; and another set with gar/amphibian/sauropsids(7)/marsupials(2), see ENSLOCP00000022432, named PRLHR_3_. The other group is also rooted by lamprey (ENSPMAP00000008908) and comprises a gar/fishes(7)/coelacanth/amphibian/sauropsids(6) set, named PRLHR_4_; and another gar/fishes(9)/coelacanth set, see ENSLACP00000014694 (removed from Ensembl subsequent releases). Three homologues of the mammalian receptors have been cloned in chicken^66^, presumably PRLHR_2–4_.

In the neuropeptide Y receptor family, three unannotated receptors NPY_6_, NPY_7_, and NPY_8_ have been described previously^67–70^.

#### AVPR; OXTR; NPSR; GNHR

Three clusters of vasopressin (AVPR) receptor genes are found: one with gar/amphibian/sauropsids(6), see ENSLOCP00000001209, named AVPR_2B_; one comprised of fishes(4) genes, see ENSDARP00000119491, named AVPR_2l_; and one with gar/fishes(14)/coelacanth, see ENSLOCP00000013628, named AVPR_2C_. Presumably AVPR_2l_ and AVPR_2C_ have been previously reported and named respectively V2-like and V2B^71^.

We found several interesting gonadotropin-releasing hormone receptors (GNRHR) groups, most including lamprey sequences at their root, whose annotation will require further study since Ensembl.R92 has clarified some of the branchings, but not all. The first contains gar/fishes(27)/coelacanth/amphibian, see ENSLOCP00000017682. The second includes fishes(16)/coelacanth/amphibian/sauropsids(6), see ENSLOCP00000010658. The remaining groups are coelacanth/amphibian/reptile(1), reptiles(5), and placental(31)/marsupial(3) mammals(see ENSMEUP00000014678). The evolution of GNRH receptors has been previously studied^72^ and four zebrafish GNRHR previously cloned^73^.

#### GRPR; BRS3; NMBR; EDNRA,B; GPR37,37L

A group constituted of gar/fishes(8)/coelacanth/amphibian/sauropsids(7)/platypus, see ENSLOCP00000018439, likely corresponds to a previously reported endothelin receptor, EDNR_B2_^74^.

#### TACR1,2,3; PROKR1,2; GPR83; GPR165

Ensembl.R92 resolves Pgr_15l_ as a new orphan present in many species: gar/fishes(8)/coelacanth/amphibian/sauropsids(6)/mammals(14 genes from rodents/rabbit), see ENSLACP00000018091. Ensembl.R95 reveals a new subtype of GPR_83_, annotated GPR_83L_, and Pgr_15l_to be a mammalian duplicate of GPR_165_.

### Chemokine (CHEM) receptors

**Figure 4:**
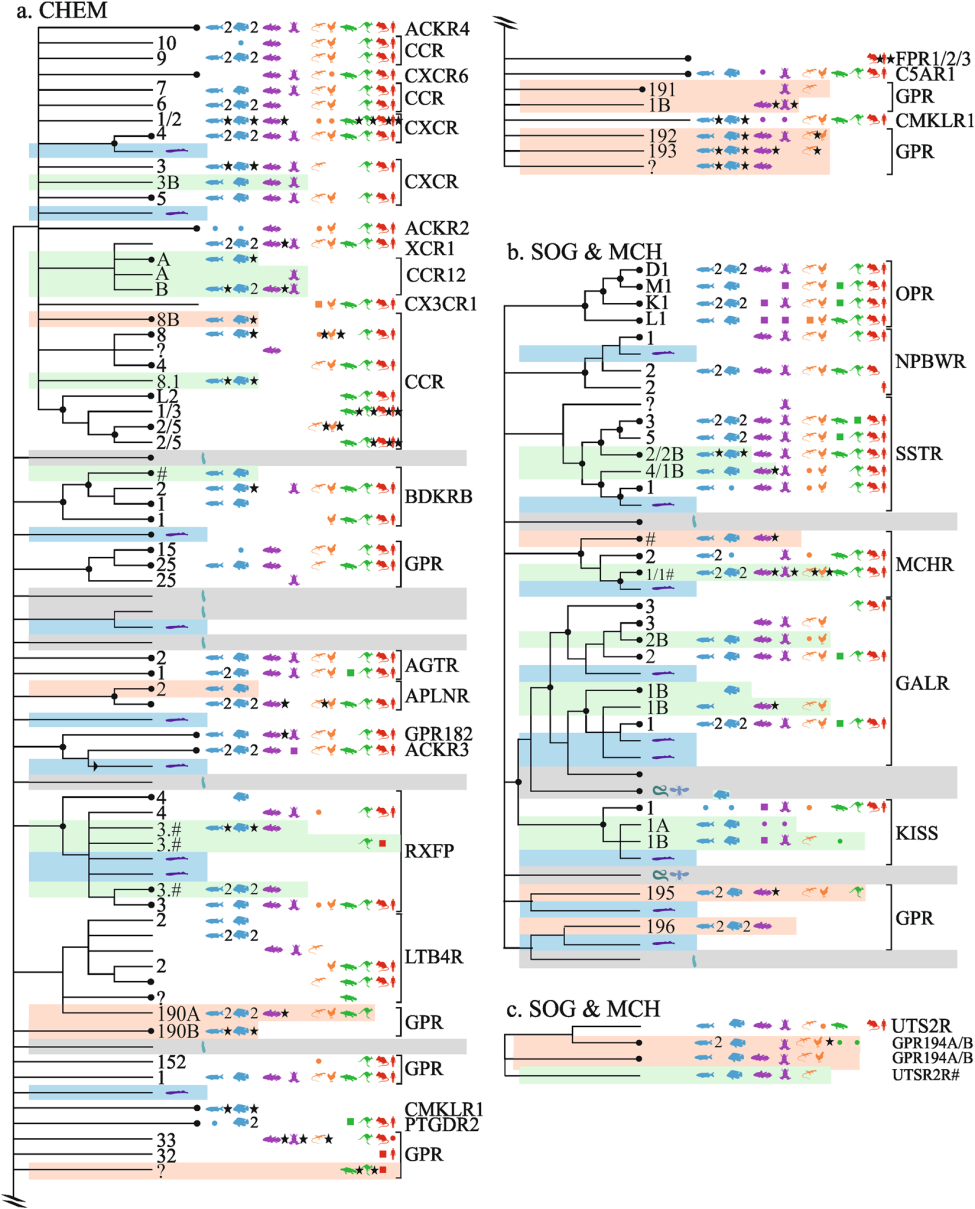
Overview of vertebrate GPCR clades mapped onto neighbor-joining trees of the γ-branch. (**a**) CHEM (**b**,**c**) SOG and MCH receptors. Caption otherwise similar to Fig. 1.

In CHEM, we report eight novel receptors, of which four are likely orphans, one divided into two subtypes (Fig. 4a). The assignment of receptors in CHEMS is difficult due to a fast evolution rate and numerous internal duplications, and additional subtypes will certainly be defined when more data becomes available. Evolution of the chemokine system has been comprehensively studied^75^.

#### LTB4R, FPR1,2,3, GPR32, GPR33, CMKLR1, C5AR1, C3AR1, PTGDR2, GPR1, GPR152

Near the leukotriene-4 receptors (LTB_4_Rs) we suggest a new orphan receptor GPR_190_, composed of two (or three) subtypes. A set of gar(duplicated)/fishes(16)/coelacanth(duplicated)/sauropsids(7) genes, see ENSLOCP00000021707, was named GPR_190A_. The second subtype is composed of gar(duplicated)/fishes(32,internal duplications)/marsupials(2), see ENSLOCP00000021705, and named GPR_190B_. A lonely platypus sequence (ENSOANP00000021820), complete with respect to its GPCR signature, may represent a third subtype. Two sets of genes near LTB_4_R_1/2_ (in Ensembl.R92) may be orthologues of existing human receptors (sets removed in E.95). Three LTB_4_R have been identified in zebrafish^76^.

The branching near the formyl peptide receptors FRP_1,2,3_ and GPR_32_ is especially blurred, and Ensembl.R92 helps understand the recent history. FRP_1,2,3_ have arose through local duplications, and marsupial(3) sequences, see ENSSHAP00000001972, are likely orthologous to the FRP_1,2,3_ set. GPR_32_ is likely rooted by seven monotreme and marsupial genes, see ENSOANP00000022386; and the FRP_1,2,3_-GPR_32_ group is likely rooted by a sequence from turtle, see ENSPSIP00000009727.

Near GPR_1_ (in Ensembl.R92), a complex cluster contains four sets of coelacanth genes (some duplicated) branching with two sets of fishes and one set (9 duplicates) of amphibian genes, see ENSLACG00000005198. This group was named GPR_1B_.

Near GPR_33_ and the chemerin chemokine-like receptor 1 (CMKLR_1_), taking shape in Ensembl.R92, there is a set comprised of fishes(2)/amphibian/reptile(lizard) genes, see ENSXETP00000062533, that was named GPR_191_. Nearby is another orphan, with several subtypes, named GPR_193_ and composed of: coelacanth/reptile(lizard), see eg ENSLACP00000014297; gar/fishes(20, internally duplicated), see ENSLOCP00000021780; isolated genes from lizard (ENSACAP00000004236) and coelacanth (ENSLACP00000021116). There is also a large set of gar/fishes (31 genes, see si:dkey-117a8.4) of ambiguous positioning. Nearby, a set of gar/fishes(26, internal duplications) genes, see ENSLOCP00000022270, was named GPR_192_; GPR_192_ may be divided into subtypes since it is branched to coelacanth/sauropsid (5) and amphibian/reptiles(5) sets, indicating an internal duplication in tetrapods.

#### CCR1,2,3,4,5,6,7,8,9,10; ACKR2,4; CXCR1,2,3,4,5,6; CX3CR1; XCR1

Three sets of fish genes were difficult to assign. Two of these sets are resolved in Ensembl.R92 and tentatively assigned to the fishes orthologous to CCR_8_, see ENSLOCP00000022371, as well as to a fish-specific clade containing gar/fishes(8), see ENSLOCP00000000990, named CCR_8B_. A third group comprising gar(duplicated)/fishes(14)coelacanth, see ENSLOCP00000022422, is located at the root of the mammalian CCR_1,2,3,4,5_ and CX3CR_1_ and probably correspond to orthologues of CCR_2_ and CCR_5_. We also identified already reported fish-specific receptors, CXCR_3B_ characterized in common carp^77^ (ENSLACP00000016977) and CCR_12A,B_ identified in orange-spotted grouper (see ENSLOCP00000022265)^78^.

#### AGTR1,2; BDKRB1,2; GPR15,25,182; APLNR; ACKR3; RXFP3,4

The bradykinin receptor B2 (BDKRB_2_) has an additional set gar/fishes(6) genes, probable result of a local duplication, see ENSLOCP00000021801. A set of gar/fishes(20)/coelacanth (also a fragmental wallaby sequence), see ENSLACP00000020198, may be paralogous to the apelin receptor (APLNR). A second group of fishes(11) nearby, see ENSAMXP00000021714, was assigned as APLNR_2_.

Human relaxin/insulin-like family peptide receptor 3 (RXFP_3_) has orthologues in fish (ENSLOCP00000021654); a paralogous group comprising gar/fishes(18)/coelacanth, see ENSLACP00000009554. Near RXFP_4_ (that has fish orthologues too) there is evidence of a new subtype comprised of gar/fishes(22, duplicated)/coelacanth/mammal(2, including one marsupial) sequences, see ENSLOCP00000022049), as well as a fish-specific set of duplicates (9, annotated rxfp3.2b). These new receptors have been named here RXFP_3.#_ pending further studies and probably have been previously described as RXFP_3–2_ and RXFP_3–3_^79,80^.

### Somatostatin-opioid-galanin (SOG) and melanin-concentrating hormone (MCH) receptors

In the SOG and MCH families we suggest five previously unreported receptor types (Fig. 4b,c), including three orphans. For a review of neuropeptide signaling systems see^81^.

#### OPRD1,-M1,-K1,-L1; NPBWR1,2; SSTR1,2,3,4,5; MCHR1,2

In the somatostatin (SSTR) receptor group, near SSTR_2_ a gar/fishes(5) group named SSTR_2B_, probable result of an internal duplication, see ENSLOCP00000021860, has been described previously as SSTR_6_^82^. Nearby, a group of fishes and coelacanth represent a new SSTR subtype, SSTR_1B_, since SSTR_4_ has lost its fishes with the exception of coelacanth^82,83^.

Branching close to MCHR_2_, there is a gar/fish(10)/coelacanth(duplicated) set, see ENSLACP00000004413. Near MCHR_1_, two new subtypes are suggested: a coelacanth/amphibian/reptile(1) set, see ENSLACP00000017963; and a larger set comprising gar/fishes(14,duplicated)/coelacanth/sauropsids(5), see ENSLACP00000009737. Mammals have five melanocortin receptors and one or two MCH receptors, zebrafish six melanocortin (extra MC5RB) and three MCH receptors; fugu four MCR (MC3R missing) and two MCHR; Two MCHR receptors have been cloned in goldfish and  four MCHR receptors have been cloned in *Xenopus tropicalis*^84^. No annotations are proposed due to the complexity of the Ensembl trees for this subfamily.

#### CCKAR; CCKBR; GPR19; KISS1R; GALR1,2,3

Near the Kisspeptin receptor 1 (KISS_1_), three unassigned sets of genes were detected. The first contains gar/amphibian, see ENSLOCP00000002856, and was named KISS_1_R_A_. The second set contains gar/fishes(10)/coelacanth/amphibian, see ENSLOCP00000003036, and was named KISS_1_R_B_; this receptor has likely been studied in ray-finned fishes^85^. A related set contains gar/coelacanth/reptiles(2), see ENSLOCP00000008391, and the tree furthermore includes fragmental sequences at the root of KISS_1_ (not discussed here).

In Ensembl.R92, the human galanin receptor 1 (GALR_1_) has two sets of paralogous fish genes (human GALR_1_ having its own fishes), rooted by a lamprey sequence (ENSPMAP00000000353). A set of gar/fishes(7)/coelacanth(2)/sauropsids(7) genes, see ENSLACP00000010695, was named GALR_1B_. Near GALR_3_, a paralogous branch with gar/fishes(10)/coelacanth/amphibian/sauropsids(4), see ENSLOCP00000001326, was named GALR_2B_. Avians have been reported to have two new types in addition to Galr_1_ and Galr_2_: GalR1, GalR1-like, GalR2-like; four galanin receptors have been isolated from European sea bass (named GalR1a, 1b, 2a, 2b)^86,87^.

Two new orphans were identified near the cholecystokinin (CCK) A and B receptors (grouped with PEP in Ensembl.R92). One is composed of fishes(8)/coelacanth(2)/sauropsids(6), see ENSLOCP00000021858, and was named GPR_195_; this receptor may have two subtypes because of coelacanth duplicates. The other is fish-specific and in Ensembl.R92 occupies its own tree, see ENSLOCP00000007402. It contains lamprey, 3R-duplicated ray-finned fishes(16), and coelacanth, and was named as GPR_196_.

#### UTS2R

Previously four urotensin 2 receptor (UTS_2_R) subtypes have been named as UTS_2_R_2–5_^82^. A set of gar/fishes(6)/coelacanth/amphibian/sauropsids(6), see ENSLACP00000012826, is orthologous to human UTS_2_R; A set from gar/fishes(9)/coelacanth/amphibian, see ENSLACP00000003013 is a probable new UTS_2_R subtype.The Ensembl.R95 gene tree suggest two subtypes of a new orphan receptor, GPR_194_: gar/fishes(2)/sauropsids/marsupials(2)/monotreme, see ENSLOCP00000022204, named GPR_194A_; and gar/fishes(10)/amphibian/birds(3), see ENSLOCP00000021948, named GPR_194B_.

### Leucine-rich repeat (LGR) and Mas-related (MRG) receptors

**Figure 5:**
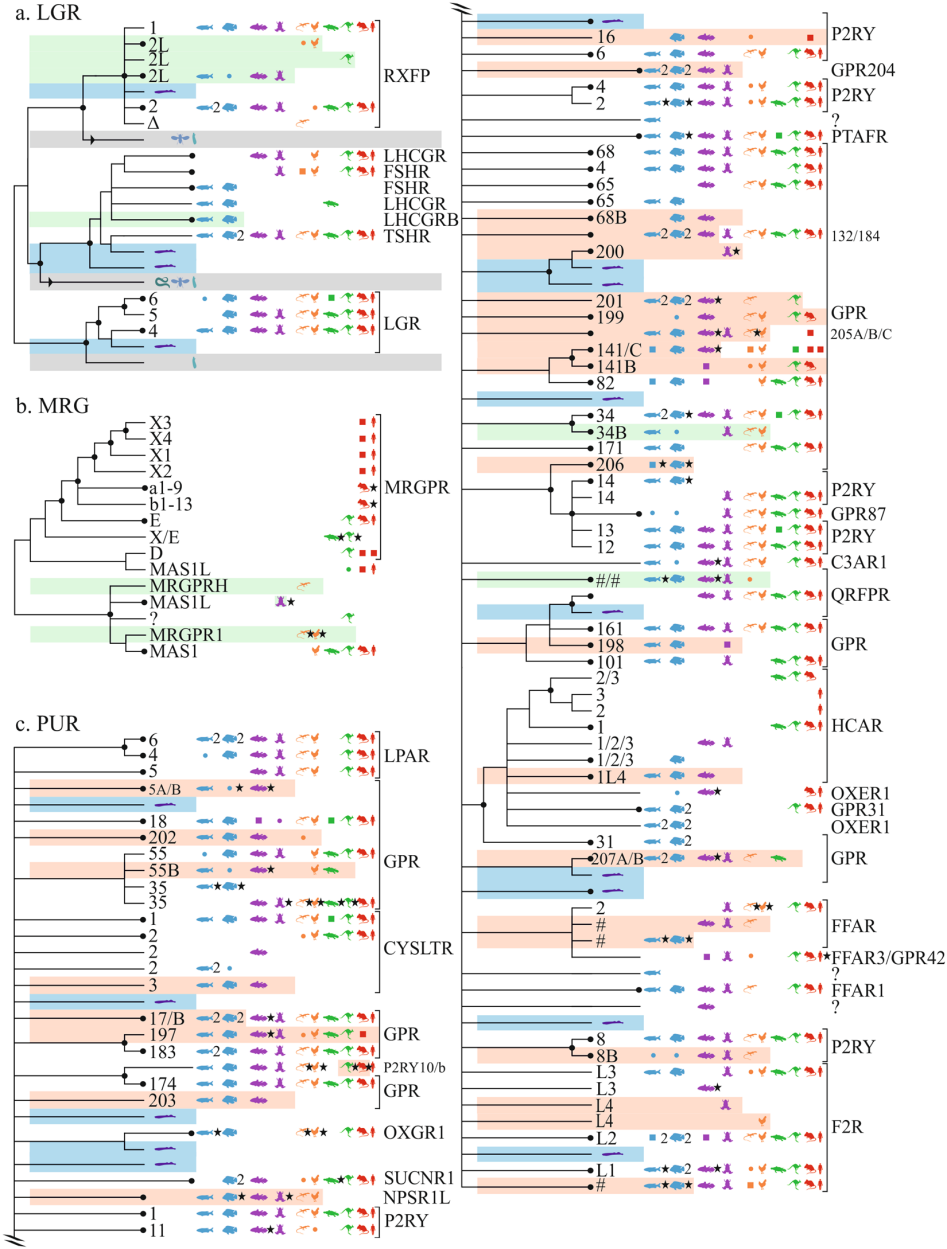
Overview of vertebrate GPCR clades mapped onto neighbor-joining trees of the δ-branch. (**a**) LGR (**b**) MGR (**c**) PUR. Caption similar to that of Fig. 1.

In the MRG and LGR families, no new vertebrate receptors were found (Fig. 5a,b).

#### LGR4,5,6; LHCGR; FSHR; TSHR; RXFP1,2

The LGRs are rooted by a set of fish genes (6, see ENSTRUP00000003361) likely orthologous to human LGR_5_. A set of gar/fishes(2)/coelacanth/amphibian/sauropsids(5)/marsupials(2) genes, see ENSLOCP00000018098, corresponds to the previously described RXFP_1B_^79^, annotated here RXFP_2L_ (marsupial and bird genes not found in R.95). A fish-specific luteinizing hormone receptor (LH), see ENSLOCP00000020088, has also been reported^88^.

#### MRGPRD; MRGPRF; MRGPRG; MRGPRE; MRGPRX1,2,3,4; MRGPRa,b; MAS1;1L

The evolution of the MAS1, MAS1 oncogene-like, and MAS-related GPR (MRG) families is complexified by numerous internal duplications. A large group of sauropsid sequences, locally duplicated, could be orthologous to the mammalian MRGPR, see ENSGALP00000047269, named here MRGPR_1_; A group of placental and marsupial mammals, see ENSSHAP00000018195, named MRGPRH. Nearby is a cluster of reptile genes, see ENSACAP00000019338, which may be orthologous or may represent a new subtype. MAS receptors have been reported to arise in amphibians^89^, and the MAS family tree is rooted by a group of amphibians, MAS_1L_, see ENSXETP00000063951. Three clusters of fish genes were positioned in Ensembl.R91 with MAS/MRGPRD but in the newer Ensembl.R92 are aggregated with GPR_1B_ (see ENSLACP00000001563, described above in the CHEM/CMKRL1 tree).

### Purine (PUR) receptors

The PUR family is the most uncharted and we identified 27 new receptors, including 12 orphans (Fig. 5c).

#### LPAR4,5,6; PTAFR; F2LR1/2/3; P2RY8; P2YR10; F2R; GPR4,18,35,55,65,68,132,174

Near LPAR_5_, a set of gar(duplicated)/fishes(2)/coelacanth(3, internally duplicated) was assigned to a new orphan probably with two subtypes, see ENSLOCP00000021495, named LPAR_5A/B_. Near PTAFR, a set of genes comprising gar/coelacanth(duplicated)/sauropsids(2)/mammalian (2, one marsupial), see ENSLOCP00000002041, was named GPR_199_.

Near LPAR_4/6_, a set with gar/fishes(4)/coelacanth/reptile(1), see ENSLOCP00000021661, was named GPR_202_. Another orphan is composed two amphibian and two lamprey sequences, see ENSXETP00000036876, named GPR_200_. Near GPR_55_, a full set of genes from gar/fishes(2)/coelacanth(duplicated)/sauropsids(5)/monotreme(1), see ENSLOCP00000021497, was named GPR_55B_.

The receptors GPR_4_, GPR_68_, GPR_65_ and GPR_132_ branch together with a new orphan, named GPR_201_, divided into two subtypes. One of these subtypes contains gar(7 duplicates)/fishes(2)/reptile(1)/marsupials(2), see ENSLACP00000013584; the other contains gar/fishes(10)/coelacanth, see ENSLACP00000020885.

Near GPR_65_ (GPR_68_ in E.95) there is a set of fishes(5)/coelacanth, see ENSLACP00000000626, was named GPR_68B_. Near GPR_132_ a set of gar/fishes(10)/coelacanth genes, see ENSLACP00000009633, was assigned to GPR_184_. Near P_2_RY_10_ and GPR_174_, a group of gar/fishes(3)/coelacanth genes, see ENSLACP00000004667, was named GPR_203_.

The coagulation factor II receptor F_2_RL_3_ is prone to local duplications, for example in spotted gar (see ENSLOCP00000014196) or coelacanth (see ENSLACP00000003039). Near F_2_RL_3_, a group of amphibian/birds(5), see ENSXETP00000005770, was named F_2_RL_4_. A set of fishes(18) paralogous to human F_2_R, see ENSAMXP00000020201, need further consideration for annotation.

Near P_2_RY_8_, a group constituted of gar/fish/coelacanth/amphibian/reptiles(2), see ENSLOCP00000021876, was named P_2_RY_8B_. Nearby a coelacanth duplicate (ENSLACP00000004454) could not be assigned. In P_2_RY_10_, a set of receptors resulting from a mammalian-specific internal duplication, see ENSMUSP00000133122, were assigned to P_2_RY_10B_.

#### GPR20,31; HCAR1,2,3; OXER1

Near GPR_20_ we suggest a new orphan, divided into two subtypes: a group of constituted of gar/fishes(6)/reptiles(3)/monotreme(1), see ENSLOCP00000001967, named here GPR_207A_; and gar/coelacanth(duplicated), see ENSLOCP00000021450, named GPR_207B_. A set of fishes(21), see ENSLOCP00000021469, is probably orthologous to the mammalian oxoeicosanoid receptor 1 (OXER1); in such case a set of fish/amphibian(2) genes, see ENSLOCP00000020331, is paralogous. The subtypes 1–3 of the hydroxycarboxylic acid receptors HCA, found only in mammals, are closely related and thus the likely result of recent duplications. Two sets of fishes are branched to the mammalian subtypes, of which gar/fishes(8)/coelacanth, see ENSLOCP00000022252, was named HCAR_1L_.

#### GPR34,82,87,171; P2RY12,13,14

P_2_RY_13_ has an extra set of fishes(3), see ENSLOCP00000002272, indicating an internal duplication. Near GPR_34_ there are two groups of sequences: one gar/fishes(41, many duplicates), see ENSLOCP00000021478, named here GPR_206_; another set with gar/fish/amphibian/sauropsids(5), see ENSLOCP00000016952, was named GPR_34B_.

#### SUCNR1; OXGR1; P2RY1,2,4,6; GPR17; GPR183; CYSTLR1,2

Branched distantly from others is a new orphan divided into three sets: two with genes from gar(2)/coelacanth/reptile, see ENSACAP00000008615, named GPR_205C_ and GPR_205B_; the second gar/fishes(9)/coelacanth/amphibian/sauropsids(4), see ENSACAP00000013831, named GPR_205A_. Near P_2_RY_1_ (NPSR_1_ in R.95) there is a cluster of genes from gar/fishes(23)/coelacanth/amphibian(triplicates)/sauropsids(7), see ENSLOCP00000022457, that we named NPSR_1L_. Near P_2_RY_6_ there are two sets of unannotated genes: a complete group with gar/fishes(3)/coelacanth/reptile(1)/mammals(21), see ENSLOCP00000021492, named here P_2_RY_16_; and, distant and rooted by duplicated lampreys (see ENSPMAP00000011278), a cluster comprised of gar/fishes(18, duplicates)/coelacanth/amphibian, see ENSLOCP00000021662, that we named GPR_204_. A set of genes from ray-finned fishes(10)/coelacanth(duplicated)/sauropsids(5)/mammals(5), including one monotreme and one marsupial), see ENSLOCP00000021877, was assigned as GPR_197_. A gar/fishes(8) specific group of genes, see ENSLOCP00000014291, is branched near GPR_17_, and we named it GPR_17B_. Another group rooted by lamprey (triplicate, ENSPMAP00000011192) comprised of gar/fishes(4)/coelacanth, see ENSPMAP00000011192, was named CYSLTR_3A_. Two other fishes (gar, cave fish) nearby open the possibility of another subtype CYSLTR_3B_.

#### FFAR_1,2,3_

Free fatty acid receptors (FFAR) have generally undergone many internal duplications. A small gene cluster from coelacanth/amphibian/reptile, see ENSLACP00000002775, is likely a new new subtype FFAR_2#_(annotation in process). A set of 32 genes from fishes, see ENSLOCP00000006360, is likely belonging to FFAR_2#_ (Ensembl.R92).

#### GPR141; GPR101,161,176

These two clusters of orphan receptors were initially aggregated with PURIN receptors but form independent groups in Ensembl.R92. A set containing gar/fishes(10)/coelacanth, see ENSLOCP00000014127, was named GPR_141C_. A set with coelacanth/sauropsids(4)/mammals(15, including two monotremes), see ENSLACP00000005080, was named GPR_141B_. A set of fishes(10)/amphibian, see ENSXETP00000035434, was named GPR_198_.

### Other families

**Figure 6:**
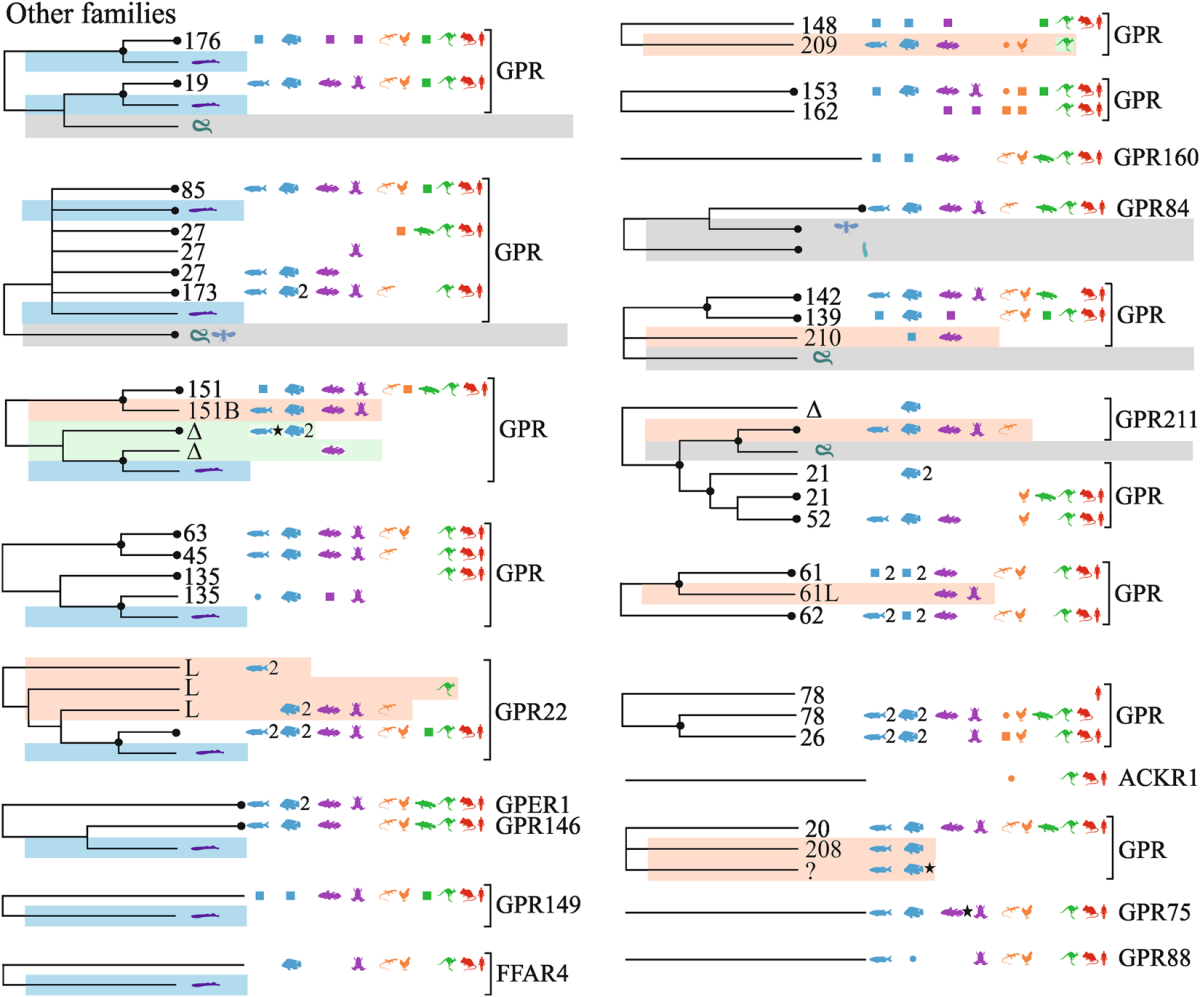
Overview of vertebrate GPCR clades mapped onto neighbor-joining trees for orphans without clear families. Caption otherwise similar to that of Fig. 1.

We grouped together receptors that we could not link to the main families, and in this group we identify seven novel receptors (Fig. 6). Interestingly, nine of the orphan groups are closely associated with a lamprey or tunicate sequence, showing an ancient origin; and there are clear grouping for some of the orphans into subtype groups.

#### GPR22

A set from gar/fishes(17,duplicated)/coelacanth/amphibian/reptile/marsupial, see ENSLOCP00000017943, is a new subtype GPR_22L_, which may divide further into different subtypes.

#### GPR139; GPR142

Three sets of genes indicate an orphan divided into three subtypes: coelacanth/fishes(4), see ENSLACP00000014196, named here GPR_210A_; gar/coelacanth, see ENSLACP00000019189 (not in R.95); gar/fishes(2)/coelacanth, see ENSLACP00000015589, named GPR_210B_.

#### GPER1

A new subtype in ray-fin fishes (8) has been previously reported, which may be a fish-specific duplicate (3R)^90^.

#### *GPR20*

An independent group of fishes(8), see ENSAMXP00000025847, was named GPR_208_; a set of fishes(10)/coelacanth/amphibian/sauropsids(7)/marsupial/monotreme genes, see ENSAMXP00000019172, is distant from GPR_20_’s placental mammals and may represent a new subtype. A group of gar/fishes(15) is also nearby in Ensembl.R92 (annotated lpar5b), see ENSLOCP00000021496.

#### GPR151

A set of fishes(9)/coelacanth/amphibian, see ENSLACP00000009338, was named GPR_151B_.

#### GPR148

A set of fish(2)/sauropsids(5)/marsupial(1), see ENSLOCP00000021506, was named GPR_209_. A second spotted gar, see ENSLOCP00000021505, may indicate another subtype.

#### GPR61

A set coelacanth/ambiphian, see ENSLACP00000015922, is likely paralogous to GPR_62. _

#### GPR21,52

A set of gar/fishes(10)/coelacanth/amphibian/lizard, see ENSLACP00000013616, named GPR_211_.

### Conservation across families

**Figure 7:**
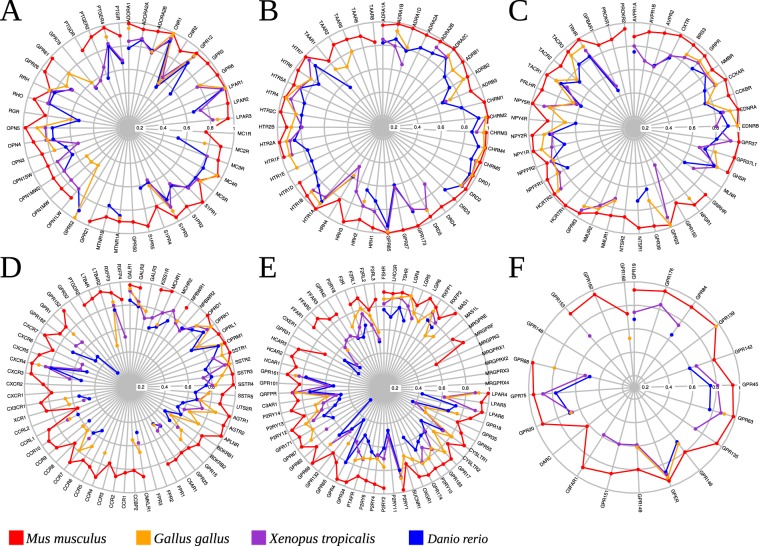
Percent sequence identity (189 aligned positions) to their human orthologues for four representative species: mouse (*Mus musculus)*, chicken (*Gallus gallus*), western clawed frog (*Xenopus tropicalis*) and zebrafish (*Danio rerio*). (**A**,**B**), α- (**C**), β- (**D**), γ- (**E**) δ- branches and (**F**) other families.

We used the set of sequences aligned across families to study the relative conservation of the subtypes across four representatives species (human, mouse, bird, amphibian and fish) (see Supporting information; 189 aligned positions). Generally, as expected the more “ancient” branches such as α-branch and β-branch are relatively conserved compared to more recent branches (χ-branch and δ-branch). In the α-branch (Fig. 7A,B), the most conserved are LPAR_1_ and CNR_1_ in MECA that share sequence identity above 90% towards their human orthologue (human-zebrafish LPAR_1_, 96%; human-mouse LPAR_1_, 99%; human-zebrafish CNR_1_ 92% and human-mouse CNR_1_ 99%). Conservation is also high in the AMIN family (Fig. 7A) where the DRD_1/2_, ADRA_2C_ and CHRM_3/4/5_ have percent sequence identities above 90% towards the human receptors. In the β-branch, in PEP GPR_22_ and HCRTR_2_ maintain sequence identity above 90% from amphibian to mouse (Fig. 7C). Few receptors of the γ-branch maintain sequence identity above 80% through species (Fig. 7D), but this is the case of OPRD_1_, OPRK_1_, GALR_1_ and SSTR_1_ (most conserved OPRM_1_, human-zebrafish 93%; human-mouse 99%). In the δ-branch conservation is less (Fig. 7E), and the most conserved are TSHR (80–93%) and P2RY_1_ (81–98%). In the unassigned receptors (Fig. 7F), GPER_1_ is the most conserved (human-zebrafish 87%; human-mouse 95%). Not surprisingly, GPR_85_ (human-zebrafish, 94%; human-mouse, 100%), an orphan receptor closely related to the amine family and named "Super Conserved Receptor" shows a very high conservation too (initially grouped with AMIN, Fig. 7A).

### Concluding remarks

The novel receptors are identified based on sequence data alone. The complete integrity of the sequences, validated by the conserved GPCR motifs, suggest that these are not pseudogenic. The groupings have been highly stable during the last five years, especially inside monophyletic groups, and most likely to remain so. Generally, the branchings in the fast-evolving PUR and CHEM families (see eg especially near FRP_1,2,3_ and GPR_32,33_), and for distant sequences such as lamprey, have been the most rearranged over the different Ensembl releases. The automated rearrangements have been nonetheless parsimonous, ie groups have been reassigned as orthologues rather than paralogues of known receptors.

This study raise numerous nomenclature issues, but in the same time sets a framework on the extent of yet-to-be studied vertebrate receptors. A difficult issue is that without establishing ligand binding preferences new sequences cannot be defined as “subtypes” or “orphan” receptors; we thus chose to keep the “subtype” denomination only for the likely cases. Importantly, the ligand binding preferences are not fully indicative of close evolutionary relationships; some receptors have acquired the same ligand specificity several times, for example α_1_-, α_2_-, and β- adrenoceptors^31,32^.

This study maps the extent of orthologues of human GPCRs to vertebrate species. Furthermore, it explores the pool of yet-to-be-studied GPCRs, suggesting at minima 69 sets of novel receptors in vertebrates not orthologous to human. The clustering data allows to group orphan receptors and thus probably brings down the number of endogenous ligands yet to be identified. Nineteen of these groups are maintained in Ensembl.R92 and thus worthy of consideration: In MECA, GPR3/6/12; in PEP, GPR83/165, EDNR/GPR37/37L1, GPR39/NTSR; in CHEM, GPR15/25, GPR182/ACK3; in PUR, GPR20/35/55, GPR101/161, P2RY10/GPR174, GPR17/183, GPR87/P2RY14, GPR31/HCAR; and in other families GPR85/27/173, GPR45/63/135, GPR153/162, GPR139/142, GPR21/52, GPR61/62, GPR26/78; for a few of these receptors an endogenous ligand has been suggested^91^. The data at the basis of this study are available online (see the Experimental section) and will continue to be improved following especially the sequencing of novel genomes and the increased coverage of existing genomes.

## Experimental Section

### Ensembl data

The Ensembl.R90 includes 89 vertebrate genomes: eleven ray-finned fishes (e.g. zebrafish, spotted gar), one lobe-finned fish, one amphibian, five birds, two reptilians, three marsupial, one monotreme and 65 eutherian mammals, as well as early vertebrates (lamprey, two tunicates) and invertebrates (one insect and one nematode). The most recent release, Ensembl.R92, includes 94 genomes.

New receptors were identified based on the branching presented on the Ensembl-generated trees. Three genomes are at the cornerstone for assessing a new receptors: coelacanth, frog, and spotted gar, and we separate them (“spotted gar” referred to simply as “gar”) from the other fishes. Spotted gar diverged from the teleost lineage before the 3R^92^). New orphan receptors were named in numerical order starting from 181, using the numbers left empty (for human genes) by the HUGO Gene Nomenclature Committee. New subtypes were identified either numerically or alphabetically.

### Guide trees

The data used to build the guide trees are based on an automatically extracted and curated set of 14.000 sequences is described as Supporting Information. This earlier data are used in this manuscript only to build guide trees and compute sequence identities in transmembrane regions.

## Electronic supplementary material

Supporting information

## Data Availability

All genomic data from the Ensembl releases is freely accessible online in few simple steps: (1) log to http://www.ensembl.org or to the Ensembl archives https://www.ensembl.org/info/website/archives/index.html (2) query the name of the receptor of interest or the provided codes; (3) select (usually) the first Gene hit on the list; (4) click on “gene tree” in the left-hand panel; (5) browse the tree data: in particular, branches can be expanded and sequences visualized. The sequence data used to construct the guide trees is accessible upon request. Note added following acceptance: The genes identified in this manuscript have been submitted to the HGNC, and will be submitted to the MGNC, RGNC, XNC,  and ZNC nomenclature committees on publication.
