# Supplementary material for: Cartography of rhodopsin-like G protein-coupled receptors across vertebrate genomes
Source: Sci Rep. 2019 May 7;9:7058. doi: 10.1038/s41598-018-33120-8 (PMC6504862; doi:10.1038/s41598-018-33120-8)
Supplement: Supplementary file 1 — Supporting information [file 41598_2018_33120_MOESM1_ESM.pdf]

Supporting Information for

Cartography of rhodopsin-like G protein-coupled receptors across vertebrate genomes

Maiju Rinne[1], Zia-Ur-Rehman Tanoli[1,2], Asifullah Khan[2], Henri Xhaard[1*]

1 Drug Research Program, Division of Pharmaceutical Chemistry and Technology, Faculty of Pharmacy, University of Helsinki, P.O. Box 56, FI-00014 University of Helsinki, Finland

2 Department of Computer and Information Sciences, Pakistan Institute of Engineering and Applied Sciences (PIEAS), P.O. 45650, Nilore, Islamabad, Pakistan. Phone: 92-51-2207381-3, Ext 3038. Fax 92-51-2208070

Table of contents:

Pages 2-4 **Procedure to manually annotate the Ensembl trees (NPFFR1 and HTR7 as examples)**

Pages 5-7 **Procedure to extract the data used to construct the guide trees and to assess the subtype conservation**

Pages 8-27 **Summary of novel and annotated vertebrate receptors without human orthologues**

Procedure to manually annotate the Ensembl tree (examples NPFFR1 and HTR7)

The annotation of the trees was essentially manual. Each annotation is based on a reasoning which accounts in particular for the annotation of orthologues, the size of the monophyletic groups, the existence of duplicates, the validity of the sequences (visually observed). The text in the manuscript is kept to minimal: due to size consideration we do not provide the reasoning beyond the identification of 147 receptor clades. Some of the annotations hinge on a single critical species, while others are more robust. The most difficult clusters of receptors were left unannotated (see text). Altogether, we aims to provide an annotation that is the most parsimonious explanation of the Ensembl.R92 data. The data is publically available and it therefore should be easy for the readers to evaluate the robustness of each new receptor clade suggested.

In the next two pages, we illustrate the reasoning of the annotation of the additional NPFFR1 and HTR7 receptors.

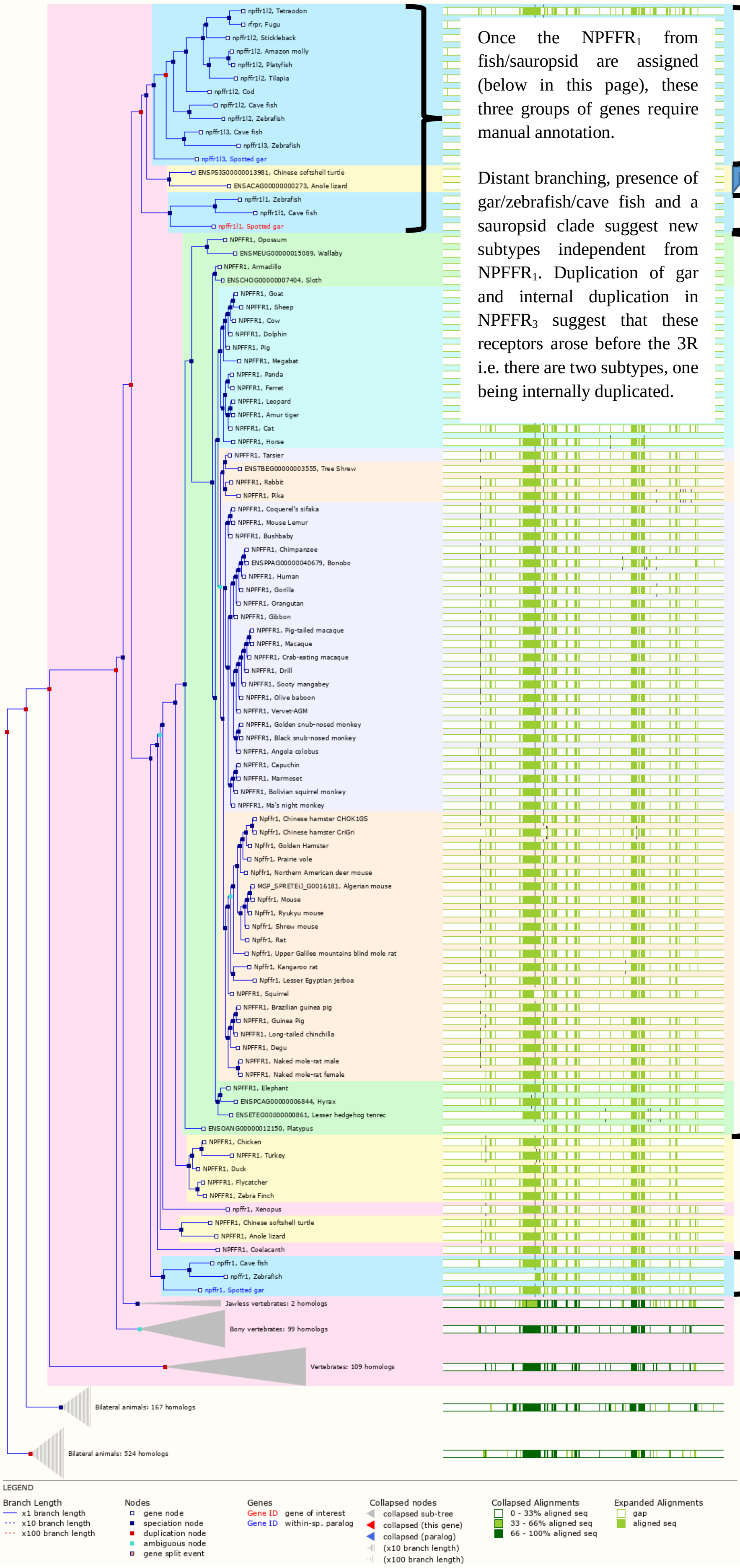

Once the $NPFFR_1$ from fish/sauropsid are assigned (below in this page), these three groups of genes require manual annotation.

Distant branching, presence of gar/zebrafish/cave fish and a sauropsid clade suggest new subtypes independent from $NPFFR_1$. Duplication of gar and internal duplication in $NPFFR_3$ suggest that these receptors arose before the 3R i.e. there are two subtypes, one being internally duplicated.

gar/fishes(11), see ENSLOCP00000019807, named $NPFFR_3$. Note the internal duplication of cave fish/zebrafish, consistent with the 3R and annotated by “2” on the guide tree

“Two reptile genes branch with either of these receptors.” The text assume the branching is not strong enough to assign them to $NPFFR_3$, but this is not the case of the guide tree.

gar/fishes(3), see ENSLOCP00000018374, named $NPFFR_4$

assigned to mammalian $NPFFR_1$

assigned to sauropsid/amphibian/coelacanth $NPFFR_1$

The Xenopus sequence should be closer to the coelacanth $NPFFR_1$ but this is a small inconsistency – it is not parsimonious to suggest an internal duplication based on the data at hand.

assigned to fish $NPFFR_1$

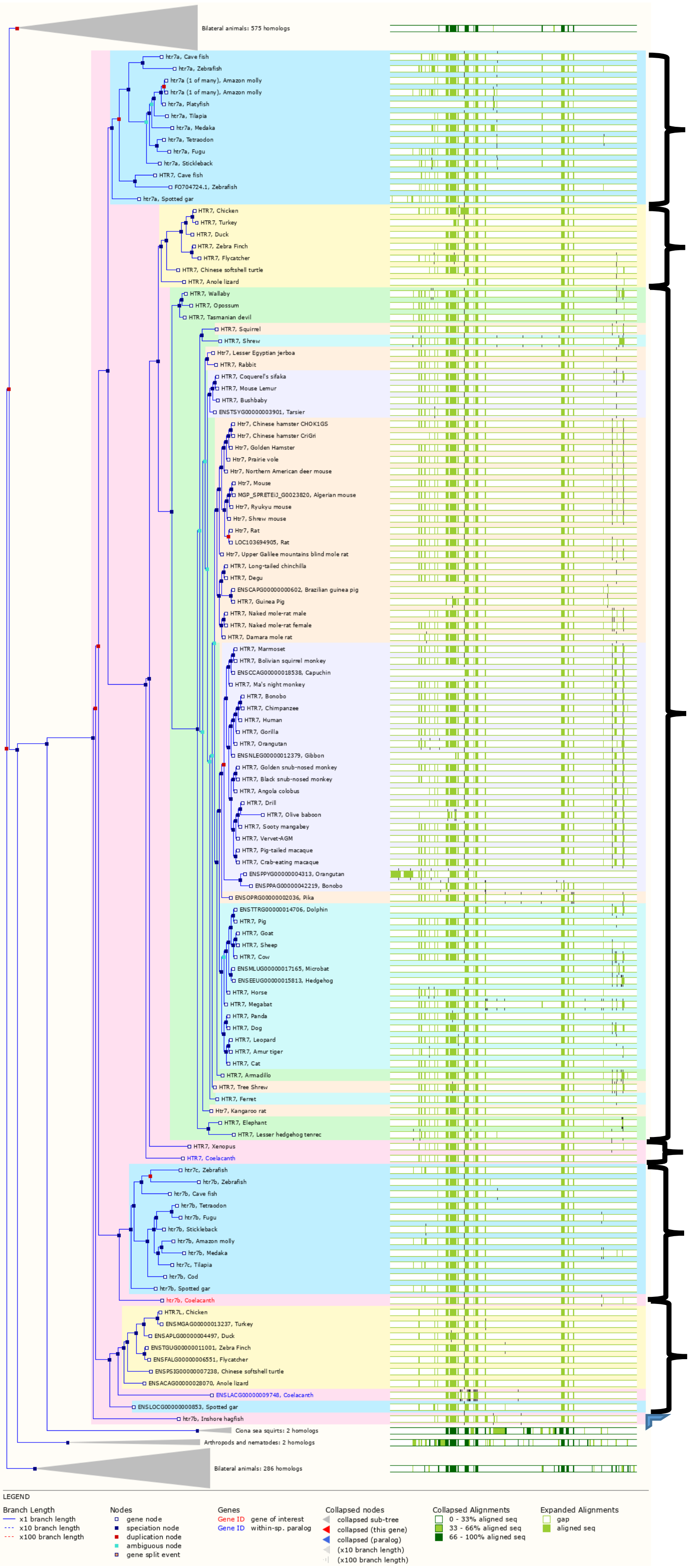

these are closest to mammalian HTR7 and have been assigned as its fish orthologues

assigned to sauropsid HTR7

assigned to mammalian HTR7

assigned to mammalian HTR7

"5-HT_{7B} (HTR7B) found in gar/fishes(10)/coelacanth, see ENSLACP00000008875, that has likely been cloned in zebrafish". Note the inconsistencies in the automated naming.

"a set of genes in gar/coelacanth/sauropsids(7), see ENSLACP00000011078, that we name 5-HT_{7C} (HTR7C)"

hagfish (R.92) is not included to our dataset and not described in this article

Procedure to extract the sequence data used to construct the guide trees

Data collection

The predicted GPCR transcripts and associated contents such as gene trees, automatically computed by in Ensembl.R67 (May 2012 release, release 67) were used as a starting point for this study (Flicek et al. 2012). The transcripts predicted in Ensembl are based on automated alignments constructed using the software MUSCLE (Flicek et al. 2012) matched to curated homologues sequences or ESTs. Gene trees are based on a consensus of five tree reconstruction methods: a maximum likelihood based on two types of distances and a neighbor-joining tree based on three types of distances.

The Ensembl.R67 includes 52 vertebrate genomes: 34 from eutherian mammals, three marsupial (*Monodelphis domestica*, *Macropus eugenii*, *Sarcophilus harrisii*), one monotreme (*Ornithorhynchus anatinus*), three birds (*Gallus gallus, Meleagris gallopavo, Taeniopygia guttata*), one reptilian (*Anolis carolinensis*); one amphibian (*Xenopus tropicalis*); nine fishes, including eight ray-finned fish such as zebrafish (*Danio rerio*) and a lobe-finned fish (*Latimeria chalumnae*). Early vertebrates are represented by the gnathostome lamprey (*Petromyzon marinus*) and by two tunicates (*Ciona intestinalis, Ciona savignyi*), the closest relative of vertebrates. Two invertebrate representatives, one for insects (*Drosophila melanogaster*), and one for nematodes (*Caenorhabditis elegans*) are also included. For comparison purpose, the most significant update in terms of species occurred around September 2015 (Ensembl.R82). The data at that time additionally includes three eutharian mammals: *Papio anubis* (olive baboon), *Ovis aries* (sheep) and *Chlorocebus sabaeus* (African green monkey), two birds: *Anas platyrhynchos* (mallard) and *Ficedula albicollis* (flycatcher), a reptile: *Pelodiscus sinensis* (Chinese softshell turtle) and three fishes: *Astyanax mexicanus* (cave fish); *Xiphophorus maculatus* (platyfish) and *Lepisosteus oculatus* (spotted gar).

The Ensembl.R67 was first queried using the list of Class A GPCR gene IDs provided by the IUPHAR (Sharman et al. 2011) completed by adding missing gene identifiers, in that case opsin GPCRs. Olfactory receptors (~460 in human) and receptors outside of class A are not included in this study. In total, about 14.000 amino acid sequences divided into 71 groups were retrieved.

Data curation

Sequence alignments of transcripts, about 14.000 in total, automatically constructed by Ensembl, were visually inspected and found to be exempt of apparent alignment errors in TM regions; this is because they are composed of closely related sequences and therefore easy to align. Individual errors

may be present in transcripts due to sequencing error or mistranslation; in particular these can take the form of a failure to properly detect the start/end codon of the gene and lead to fragmental sequences; furthermore in poorly assembled genomes two or more occurrences of one gene, resembling duplicates, may be found. Automated curation was therefore conducted. First, for each transmembrane segment we manually annotated as reference position the well-defined conserved positions in class A GPCRs (see introduction): N1.50, D2.50, R3.50, W4.50, P5.50, P6.50, P7.50. In case the amino acid at that position varied, the canonical motif at that transmembrane segment was used in its entirety to identify the equivalent position. These “pivot” positions were assigned to each of the seven TMs for each of the 71 groups of aligned sequences extracted from Ensembl, which is *de facto* equivalent to aligning all the 14000 sequences in a large multiple sequence alignment. A human receptor sequence (the first one to occur in the file) was furthermore taken as a reference for the curation of each of the 71 groups. The gene trees were further manually annotated to assign working names to all transcripts (including to give a working name to sequences without close relatives), and computational scripts used to replace Ensembl IDs with these working names in order to ease the analysis.

Secondly, taking advantage of the reference sequences and the annotated pivot positions, we kept only sequences that have at maximum four deletions (gaps) in each of the seven TM segment and a minimum of 10% sequence identity towards their reference sequences. These values allowed us to eliminate mistranslated sequences and fragments, but in the same time keeping the more distant sequences of invertebrates if any. Using a more stringent criteria (<15%) would lead to overall similar results (data not shown). Human receptors were chosen as reference since they are well characterized, appropriately named, and should not include missing regions in their amino acid sequences. The curation step left us with ~11.000 sequences to work with (Table S1). The procedure worked well and only few cases (GPR_{119}, Adenosine A3, PTGER family) we apparently eliminated too much sequences. Visual observation shows a very good quality of multiple protein sequence alignments used to build the trees, in particular of the seven “pivot” positions.

Table S1. Sequence counts for vertebrates in Ensembl.R67.

			Danio rerio		*Xenopus tropicalis*		*Gallus gallus*		*Mus musculus*		*Homo sapiens*	
	Family, abbreviation	Family	all	curated	all	curated	all	curated	all	curated	all	curated
α-branch	AMIN	Amine	177	149	39	36	42	34	53	53	43	43
	MECA	Melanocortin, EDG, Cannabinoid, Adenosine	33	31	22	20	18	14	24	22	23	21
	OPN	Opsin	42	35	22	16	15	11	9	9	12	12
	MLT	Melatonin	7	7	6	0	3	2	3	3	3	3
	PTEGR	Prostaglandin	18	9	11	7	6	4	11	6	12	7
β-branch	PEP	Peptide	69	67	48	43	43	36	44	44	42	41
γ-branch	SOG	Somatostatin, Opioid, Galanin	24	23	24	20	19	16	14	14	16	16
	CHEM	Chemokine	85	80	35	33	27	27	50	49	44	43
	MCHR	Melanin-concentrating hormone	5	5	3	3	1	1	1	1	2	2
δ-branch	LRG	Glycoprotein	7	7	7	7	7	7	7	7	7	7
	PUR	Purin	92	80	55	50	74	67	49	48	50	49
	MRG	MAS	0	0	5	4	4	3	21	20	10	9
Orphan families	Orphan families		22	15	17	16	14	8	20	20	21	21

References:

Flicek PM, Amode R, Barrell D, Beal K, Brent S, Carvalho-Silva D, Clapham P, Coates G, Fairley S, Fitzgerald S, et al**.** Ensembl 2012. *Nucleic Acids Research* 2012. 40: D84-D90.

Sharman JL, Mpamhanga CP, Spedding M, Germain P, Staels B, Dacquet C, Laudet V, Harmar AJ, NC-IUPHAR (2011). IUPHAR-DB: new receptors and tools for easy searching and visualization of pharmacological data. *Nucleic Acids Research.* 39 (Database Issue): D534-D538.

Supplementary Tables: Sequence counts in Ensembl.R91.

Probable new receptors are highlighted in orange. Note that the mouse symbol refer to non-human placental mammals in general, not to rodents in particular.

**AMIN**	Lamprey (a)	Fishes (*Actinopterygii*)				Fishes (*Sarcopterygii*)	Amphib-ian	Rep-tiles	Birds		Mammals		Comments
			x2		x2								
ADRA2D	1	4‡	y	15†	y	1	1	1	3				Expression, synteny, phylogeny and pharmacology see Ruuskanen *et al.*, 2004, 2005.
DRD2l		1	n	8†	n	1							Phylogeny, expression, synteny of four zebrafish receptors (D2 and D3a, D3b, D3c), see Boehmler *et al.*, 2004. Annotated as DRD2l and DRD4-rs. DRD2L is not a splice variant of DRD2.
DRD4-rs		2	n	6†	n	1							
DRD6		3	y	13†‡	y	1	1						
DRD8						1		2	2				Phylogeny, expression, synteny, see Yamamoto *et al.*, 2013. Named D1C, D1X, D1E in the reference.
DRD7		1	n	1†	n			1	5				
HTR4B		2	n	9†	n	1							Annotated as si:dkey-247m21.3.
HTR5C				1†	n	1			3				Possible orthologue of HTR5B.
HTR7B		3‡	n	8†	n	1							Gene expression of zebrafish orthologues of the 14 human receptors in zebrafish larvae, Sourbron *et al.*, 2016.
HTR7C						1		2	5				
HRH5	1	2	n	1†	n		1	2	5		2	2	
CHRM6		1	n	7	n								Annotated as si:ch73-151m17.5

										Cloning, phylogeny, expression: at least five genes in zebrafish named chrm1a, chrm1b, chmr3a, chrm5a and chrm5b, see Nuckels *et al.,* 2011.
TAAR?		14‡	y	109†‡	y					TAAR receptors have a very complex evolutionary history, and assigning names is difficult. See Hussain *et al*., 2009
GPR181	2			1†	n	1	1	2	5	
GPR189	4	1	n	9†	n	1	1			

(a) at the root/near the root of a group/branch/cluster of subtypes, (x2) 3R-fish specific duplication, (†) spotted gar orthologue, (‡) local duplication

Boehmler, W., Obrecht-Pflumio, S., Canfield, V., Thisse, C., Thisse, B., and Levenson, R. (2004) Evolution and expression of D2 and D3 dopamine receptor genes in zebrafish. Developmental dynamics: an official publication of the American Association of Anatomists. 230(3):481-493.

Hussain, A., Saraiva, L.R., and Korsching, S.I. (2009) Positive Darwinian selection and the birth of an olfactory receptor clade in teleosts. Proc. Natl. Acad. Sci. USA. 106(11):4313-4318.

Nuckels, R.J., Forstner, M.R., Capalbo-Pitts, E.L., García, D.M. (2011) Developmental expression of muscarinic receptors in the eyes of zebrafish. Brain Res. 1405:85-94.

Ruuskanen, J.O., Laurila, J., Xhaard, H., Rantanen, V.V., Vuoriluoto, K., Wurster, S., Marjamäki, A., Vainio, M., Johnson, M.S., Scheinin, M. (2005) Conserved structural, pharmacological and functional properties among the three human and five zebrafish alpha 2-adrenoceptors. Br J Pharmacol. 144(2):165-77.

Ruuskanen, J., Xhaard, H., Marjamäki, A., Salaneck, E., Salminen, T., Yan, Y.-L., Postlethwait, J.H., Johnson, M.S., Larhammar, D., and Scheinin, M. (2004) Identification of duplicated fourth {alpha}2-adrenergic receptor subtype by cloning and mapping of five receptor genes in zebrafish. Mol. Biol. Evol.. 10:14-28.

Sourbron, J., Schneider, H., Kecskés, A., Liu, Y., Buening, E.M., Lagae, L., Smolders, I., de Witte, P.A. (2016) Serotonergic Modulation as Effective Treatment for Dravet syndrome in a Zebrafish Mutant Model. ACS Chemical Neuroscience. 7(5):588-98.

MECA	Lamprey (a)	Fishes (Actinopterygii)				Fishes (Sarcopterygii)	Amphib-ian	Rep-tiles	Birds	Mammals			Comments
	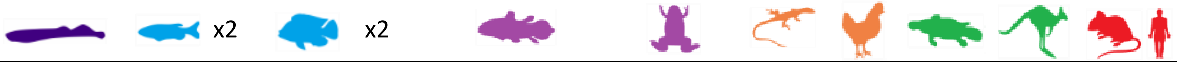		x2		x2								
S1PR6		3‡	y	15†	y	1		1					Seven zebrafish S1PR isolated, see Hisano *et al.*, 2015
S1PR7		2	n	9†	n	1							
GPR185		4	y	6†	n	1	1	1	2	1	1		Cloned in X. Laevis, named GPRx, Rios-Cardona 2008. Named GPR185 in Nader *et al*. 2014
GPR186		2	n	7	n								Annotated as GPR186.
GPR119B				1†	n			2	3				
GPR187A	1	2	n	8†	n	2‡							Fishes annotated as si:dkey-206f10.5.
GPR187B				2	n	1	1	2					

(a) at the root/near the root of a group/branch/cluster of subtypes, (x2) 3R-fish specific duplication, (†) spotted gar orthologue, (‡) local duplication

Hisano, Y., Inoue, A., Taimatsu, K., Ota, S., Ohga, R., Kotani, H., Muraki, M., Aoki, J., Kawahara, A. (2015) Comprehensive analysis of sphingosine-1-phosphate receptor mutants during zebrafish embryogenesis. Genes to cells: devoted to molecular & cellular mechanisms. 20(8):647-58.

Nader, N., Dib, M., Daalis, A., Kulkarni, R.P., Machaca, K. (2014) Role for endocytosis of a constitutively active GPCR (GPR185) in releasing vertebrate oocyte meiotic arrest. Dev Biol. 395(2):355-66.

**OPN**	Lamprey (a)	Fishes (Actinopterygii)				Fishes (Sarcopterygii)	Amphib-ian	Rep-tiles	Birds	Mammals			Comments
	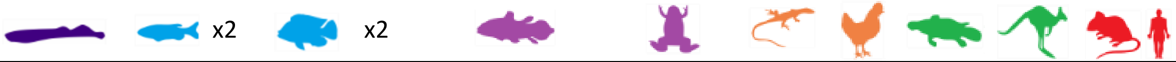		x2		x2								
RHO2		5‡	n	18†‡	n	1		2	6‡				Some species annotated as opn1mw1-4. See Chinen *et al.,* 2003
OPN1SW2		2	n	10†‡	n	1	1	2					Some species annotated as OPN1SW2. Chinen *et al.,* 2003
Pinopsin				1†	n	1	1	2	5				
VA-opsin	1	4	y	9†	n	1	1	2	5				
Parietopsin		2	n	6	n		1	1					
Parapin-opsin	1	3	y	15†	y			1					
OPN3B		4	y	16†‡	y		1	1	1				Some species annotated as tmtopsb, tmtops2a/b, tmtops3a/b.
OPN3C		3	y	14†	y		1						
OPN3D		4	y	10†	y			2	5		1		
OPN4B		2	n	6†	n								Melanopsins annotated as opn4.1 or opn4xa/b. Sequence similarity, chromosomal localization, phylogeny, see Bellingham *et al.*, 2006.
OPN4C		3	y	17†	y	1	1	2	5				
OPN5L2		5‡	y	16†	y		1	2	1				Tissue distribution, gene synteny, phylogeny of OPN5 see Tomonari *et al.,* 2008. Sato *et al.*, 2016. See names in parenthesis
OPN5L1		2	n	9†	n	1	1	2	3				
OPN5L3a (OPN8b)		1	n	8†	n		1	2	5				
OPN5L3b		1	n	6†‡	n								
OPN5L3c (OPN8c)		1	n	7†	n								
OPN5L4a (OPN6a)		3	y	16†	y	1	1	2	5	1			

OPN5L4b (OPN6b)	2	n	1†	n	1	1
OPN5L5 (OPN9)	2	n	4†	n		

(a) at the root/near the root of a group/branch/cluster of subtypes, (x2) 3R-fish specific duplication, (†) spotted gar orthologue, (‡) local duplication

Bellingham, J., Chaurasia, S.S., Melyan, Z., Liu, C., Cameron, M.A., Tarttelin, E.E., Iuvone, P.M., Hankins, M.W., Tosini, G., and Lucas, R.J. (2006) Evolution of Melanopsin Photoreceptors: Discovery and Characterization of a New Melanopsin in Nonmammalian Vertebrates. PLoS Biology. 4(8):e254.

Chinen, A., Hamaoka, T., Yamada, Y., Kawamura, S. (2003) Gene duplication and spectral diversification of cone visual pigments of zebrafish. Genetics. 163(2):663–675.

Sato, K., Yamashita, T., Haruki, Y., Ohuchi, H., Kinoshita, M., Shichida, Y. (2016) Two UV-Sensitive Photoreceptor Proteins, Opn5m and Opn5m2 in Ray-Finned Fish with Distinct Molecular Properties and Broad Distribution in the Retina and Brain. PLoS One. 11:e0155339.

Tomonari, S., Migita, K., Takagi, A., Noji, S., Ohuchi, H. (2008) Expression patterns of the opsin 5–related genes in the developing chicken retina. Dev Dyn. 237: 1910–1922.

**PTGER**	Lamprey (a)	Fishes (Actinopterygii)				Fishes (Sarcopterygii)	Amphib-ian	Rep-tiles	Birds	Mammals	Comments
	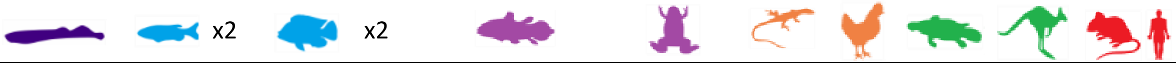		x2		x2						
PTGER5	1			8†	n	1					Five contractile and one inhibitory prostanoid receptors in zebrafish. Iwasaki *et al.,* 2013.
GPR204	1	2	n	10†‡	n	1					

(a) at the root/near the root of a group/branch/cluster of subtypes, (x2) 3R-fish specific duplication, (†) spotted gar orthologue, (‡) local duplication

Iwasaki, R., Tsuge, K., Morimoto, K., Inazumi, T., Kawahara, O., Kawahara, A., Tsuchiya, S., Sugimoto, Y. (2013) Molecular and pharmacological characterization of zebrafish 'contractile' and 'inhibitory' prostanoid receptors. Biochem Biophys Res Commun. 438(2):353-8.

**MLT**	Lamprey (a)	Fishes (Actinopterygii)				Fishes (Sarcopterygii)	Amphib-ian	Rep-tiles	Birds	Mammals				Comments
	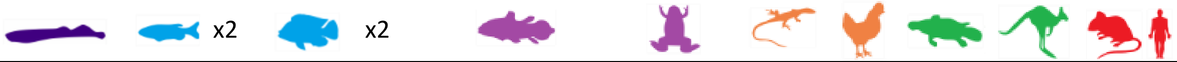		x2		x2									
MTNR1al	1	2	n	8†‡	n		1	2		1				Six Mtnr1 proteins in zebrafish (immunochemistry). See Villarreal *et al*., 2017.Some species annotated as MTNR1al.
MTNR1C		2	n	9†	n	1	2‡	2	5	1				Some species annotated as MTNR1c. See Reppert *et al*., 1995
MTNR1D		2	n	2‡	n									Fishes annotated as mtnrba or mtnr1al.

(a) at the root/near the root of a group/branch/cluster of subtypes, (x2) 3R-fish specific duplication, (†) spotted gar orthologue, (‡) local duplication

Reppert, S.M., Weaver, D.R., Cassone, V.M., Godson, C., and Kolakowski, L.F., Jr. (1995) Melatonin receptors are for the birds: molecular analysis of two receptor subtypes differentially expressed in chick brain. Neuron. 15:1003-1015.

Villarreal, M.A., Biediger, N.M., Bonner, N.A., Miller, J.N., Zepeda, S.K., Ricard, B.J., García, D.M., Lewis, K.A. (2017) Determining Zebrafish Epitope Reactivity to Commercially Available Antibodies. Zebrafish. 14(4):387-389.

**PEP**	Lamprey (a)	Fishes (Actinopterygii)				Fishes (Sarcopterygii)	Amphib-ian	Rep-tiles	Birds	Mammals			Comments
	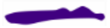	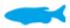	x2	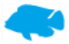	x2	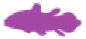	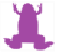	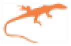	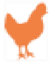	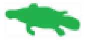	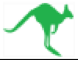	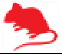 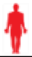	
EDNRC		1	n	8†	n	1	1	2	5	1			Phylogeny, chromosomal mapping see Hyndman *et al.,* 2009
MLNR2		1	n	9†	n	1	1						Fishes may be paralogous.
MLNR3						1	1	2					
GHSR2		2	n	9†	n	1							Four mRNA identified in Goldfish Kaiya *et al.*, 2010
GHSR3		3	y	8	n								
NMUR3		2	n	9†	n	1							NMUR3 annotated.
TRHR2		2	n	9†‡	n	1	1	1		1	1	18	Bidaud *et al*., 2004<br>Mekuchi *et al.*, 2011
TRHR3		2	n	10†‡	n	1	1	2	6‡		3	11	
GNRHR3	1	3	y	25†‡	y	1	1		5				Unstable branching in Ensembl.R92
GNRHR2	1					1	1	1			3	31	Phylogenetic and syntenic analysis suggest four GnRH receptors each divided in two groups in zebrafish, see Tello *et al.,* 2008. Chromosomal mapping, see Kim *et al.,* 2011. Named GnRHR1, GnRHR2, GnRHR3, GnRHR4.
GNRHR4	1	4	y	12†	y	1	1	2	4				
NPFFR3		4	y	8†	n			2					Ambiguous branching of reptile sequences.
NPFFR4		2	n	1†	n								
AVPR3				1†			1	1	5				
AVPR4		2	n	12†	y	1							A phylogenetic analysis, see Daza *et al.,* 2012, suggests V1A, V1B, v2A, V2B, V2-like, OT-R, in teleost fish.<br>Some fishes annotated as si:dkey-178o16.4
AVPR2l				4	n								Annotated as AVPR2l.
Pgr15l		1	n	8†		1	1	1	6‡			15	

PRLHR2		4	y	13†	y	1	1	2	3				Three homologues of mammalian PrRP cloned from chicken and named cPrRPR1, cPrRPR2, cC-RFaR, see Wang *et al.* 2012.
PRLHR3	1			1†	n		1	2	5		2		PRLHR2/3 might form an orphan receptor.
PRLHR4	1	2	n	6†	n	1	1	2	4				Annotated as prlh2r. PRLHR4/5 might form an orphan receptor.
PRLHR5		1	n	9†	n	1							Annotated as si:dkey-202l22.3. PRLHR4/5 might form an orphan receptor.
NPY7R		2	n	9†	n	1	1	2	5	1			Fishes, coelacanth and xenopus annotated as npy7r. See Fredriksson *et al*., 2004, Larhammar and Bergqvist, 2013
NPY8R		3	y	15†	y	1	1	1					See Fredriksson *et al*., 2006, Larhammar and Bergqvist, 2013
NPY6R				1†	n	1		2	5	1	1	19	Some of the placental mammals annotated as npy6r. Weinberg *et al*., 1996
GPR205				4†	n	1	1						

(a) at the root/near the root of a group/branch/cluster of subtypes, (x2) 3R-fish specific duplication, (†) spotted gar orthologue, (‡) local duplication

Bidaud, I., Lory, P., Nicolas, P., Bulant, M., Ladram, A. (2002) Characterization and functional expression of cDNAs encoding thyrotropin-releasing hormone receptor from *Xenopus laevis*. Identification of a novel subtype of thyrotropin-releasing hormone receptor. Eur J Biochem 269: 4566–4576.

Daza, D.O., Lewicka, M., and Larhammar, D. (2012) The oxytocin/vasopressin receptor family has at least five members in the gnathostome lineage, inclucing two distinct V2 subtypes. Gen. Comp. Endocrinol.. 175(1):135-143.

Fredriksson, R., Larson E.T., Yan, Y.-L., Postlethwait, J.H., and Larhammar, D. (2004) Novel neuropeptide Y Y2-like receptor subtype in zebrafish and frogs supports early vertebrate chromosome duplications. Journal of molecular evolution. 58(1):106-114.

Fredriksson, R., Sjodin, P., Larson, E.T., Conlon, J.M., and Larhammar, D. (2006) Cloning and characterization of a zebrafish Y2 receptor. Regulatory peptides. 133(1-3):32-40.

Kaiya, H., Miura, T., Matsuda, K., Miyazato, M., Kangawa, K. (2010) Two functional growth hormone secretagogue receptor (ghrelin receptor) type 1a and 2a in goldfish, Carassius auratus. Mol Cell Endocrinol. 327(1-2):25-39.

Kim, D.-K., Cho, E.B., Moon, M.J., Park, S., Hwang, J.-I., Kah, O., et al. (2011) Revisiting the evolution of gonadotropin-releasing hormones and their receptors in vertebrates: secrets hidden in genomes. Gen. Comp. Endocrinol. 170: 68–7810.

Larhammar, D., and Bergqvist, C.A. (2013) Ancient Grandeur of the Vertebrate Neuropeptide Y System Shown by the Coelacanth Latimeria chalumnae. Frontiers in Neuroendocrine Science. 7:27.

Mekuchi, M., Saito, Y., Aoki, Y., Masuda, T., Iigo, M., Yanagisawa, T. (2011) Molecular cloning, gene structure, molecular evolution and expression analyses of thyrotropin-releasing hormone receptors from medaka (Oryzias latipes). Gen Comp Endocrinol. 170(2):374-80.

Tello, J.A., Wu, S., Rivier, J.E., and Sherwood, N.M. (2008) Four functional GnRH receptors in zebrafish: Analysis of structure, signaling, synteny and phylogeny. Integrative and Comparative Biology. 48(5):570-587.

Wang, Y., Wang, C.Y., Wu, Y., Huang, G., Li, J., and Leung, F.C. (2012) Identification of the Receptors for Prolactin-Releasing Peptide (PrRP) and Carassius RFamide Peptide (C-RFa) in Chickens. Endocrinology. 153(4):1861-1874.

Weinberg, D.H., Sirinathsinghji, D.J., Tan, C.P., Shiao, L.L., Morin, N., Rigby, M.R., Heavens, R.H., Rapoport, D.R., Bayne, M.L., Cascieri, M.A., Strader, C.D., Linemeyer, D.L., MacNeil, D.J. (1996) Cloning and expression of a novel neuropeptide Y receptor. J Biol Chem. 271(28):16435-8.

CHEM	Lamprey (a)	Fishes (Actinopterygii)				Fishes (Sarcopterygii)	Amphib-ian	Rep-tiles	Birds	Mammals			Comments
			x2		x2								
CCR2/5B								4‡	8‡				Difficult to assign. Devries *et al.*, 2006
CCR8B		2	n	7†	n								Fish-specific clade. Tentative assignment. Annotated si:cabz01093077.1
CCR12		5‡	n	11†‡	n								
CXCR3B		2	n	7	n	1	1						Fishes annotated as cxcr3.2. Devries *et al.*, 2006
XCR1B		1	n	10†‡	n		1						Devries *et al.*, 2006
XCR1C		4‡	y	27†‡	y	3‡	1						Some species annotated as ccr12a/b. Devries *et al.*, 2006
BDKRB3		2	n	5†‡	n								Duplicated in cod.
APLNR2		2	n	9‡	n								Another set containing gar/fishes/coelacanth nearby may belong to a third subtype.
RXFP3B		4	y	15†	y	1							Annotated as rxfp3.2a/b. Good *et al.*, 2012
RXFP4B		6‡	y	25†‡	y	1				1	1		Includes a paralogous branch of fishes.<br>Annotated as rxfp3.3a/b. Good *et al.*, 2012
LTB4R3		5‡	y	15	y	1	1	1					Probable orthologue of LTB4R1<br>See Okuno *et al.*, 2015
LTB4R4		2	n	8‡	n								Possible orthologue of LTB4R2
GPR190A		4	y	14†	y	2‡		2	5	1	1		Fishes annotated as si:dkey-148a17.5/6. Branched near LTB4R.

GPR190B	13‡	n	25†‡	n					Some sequences annotated as si:ch73-113g13.1/2/3/4. Branched near LTB4R.
GPR191					3‡	9‡			Probably divided into subtypes
GPR192	2	n	26†‡	n	1	1	6‡	5	Probably divided into subtypes<br>Fishes annotated as fpr1.
GPR207	1	n	1†	n		1	1		Two fishes added to this set in Ensembl.R92
GPR208	6‡	n	14†‡	n	2‡		2‡		Probably divided into subtypes.<br>Unassigned set of fishes (si:dkey-117a8.4) nearby

(a) at the root/near the root of a group/branch/cluster of subtypes, (†) spotted gar orthologue, (‡) local duplication

Devries, M.E., Kelvin, A.A., Xu, L., Ran, L., Robinson, J., and Kelvin, D.J. (2006) Defining the origins and evolution of the chemokine/chemokine receptor system. Journal of immunology (Baltimore, Md. : 1950). 176(1):401-415.

Good, S., Yegorov, S., Martijn, J., Franck, J., and Bogerd, J. (2012) New insights into ligand-receptor pairing and coevolution of relaxin family peptides and their receptors in teleosts. International Journal of Evolutionary Biology. 2012:310278.

Okuno, T., Ishitani, T., Yokomizo, T. (2015) Biochemical Characterization of Three BLT Receptors in Zebrafish. PLoS One. 10:e0117888.

**SOG & MCH**	Lamprey (a)	Fishes (Actinopterygii)				Fishes (Sarcopterygii)	Amphibian	Reptiles	Birds	Mammals			Comments
	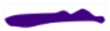	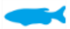	x2	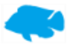	x2	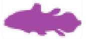	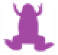	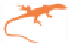	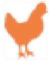	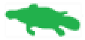	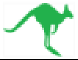	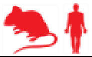	
SSTR2B		1	n	5†	n								Hagemeister *et al.*, 2010, Tostivint *et al.*, 2014
SSTR4B		3	n	3†	n	1							
MCHR3		2	n	9†	n	2							
MCHR1B						1	1	1					Kobayashi *et al.*, 2015
MCHR1C		4	y	11†	y	1		1	4				
GALR4		2	n	9†	n	1	1	1	3				Ho *et al.*, 2012
GALR5	1	2	n	7†	n	2‡		2	5				Ho *et al.*, 2011
KISS2R		1	n	2†	n	1	2‡						Branching inconsistent in Ensembl.R92. Fishes annotated as kiss1rb. Pasquier *et al.*, 2014
KISS3R		2	n	10†‡	n	2‡	1	2		1			Branching inconsistent in Ensembl.R92. May be divided in two subtypes. Pasquier *et al.*, 2014
UTS2RB		2	n	1†	n			2	1	1	2		Tostivint *et al.*, 2014
UTS2RC		2	n	9†	n		1		3				Tostivint *et al.*, 2014
UTS2Rd		1	n	6†	n	1	1	2	4				Tostivint *et al.*, 2014
UTS2RE		2	n	8	n	1	1	1					Most of the species annotated as s:idkey-27n14.1. Tostivint *et al.*, 2014

GPR193	1	3	y	9†	n	2		1	5	2
GPR194	1	3	y	15†	y	1				

(a) at the root/near the root of a group/branch/cluster of subtypes, (†) spotted gar orthologue, (‡) local duplication

Hagemeister, A.L., Kittilson, J.D., Bergan, H.E., Sheridan, M.A. (2010) Rainbow trout somatostatin receptor subtypes SSTR1A, SSTR1B, and SSTR2 differentially activate the extracellular signal-regulated kinase and phosphatidylinositol 3-kinase signaling pathways in transfected cells. J Mol Endocrinol. 45(5):317-27.

Ho, J.C., Jacobs, T., Wang, Y., Leung, F.C. (2012) Identification and characterization of the chicken galanin receptor GalR2 and a novel GalR2-like receptor (GalR2-L). Gen Comp Endocrinol. 179(2):305-12.

Ho, J.C., Kwok, A.H., Zhao, D., Wang, Y., Leung, F.C. (2011) Characterization of the chicken galanin type I receptor (GalR1) and a novel GalR1-like receptor (GalR1-L). Gen Comp Endocrinol. 170(2):391-400.

Kobayashi, Y., Hamamoto, A., Hirayama, T., Saito, Y. (2015) Molecular cloning, expression, and signaling pathway of four melanin-concentrating hormone receptors from Xenopus tropicalis. Gen Comp Endocrinol. 212:114-23.

Martins, R.S., Pinto, P.I., Guerreiro, P.M., Zanuy, S., Carrillo, M., Canário, A.V. (2014) Novel galanin receptors in teleost fish: identification, expression and regulation by sex steroids. Gen Comp Endocrinol. 205:109-20.

Pasquier, J., Kamech, N., Lafont, A.G., Vaudry, H., Rousseau, K., Dufour, S. (2014) Molecular evolution of GPCRs: Kisspeptin/kisspeptin receptors. J Mol Endocrinol. 52(3):T101-17.

Tostivint, H., Ocampo Daza, D., Bergqvist, C.A., Quan, F.B., Bougerol, M., Lihrmann, I., Larhammar, D. (2014) Molecular evolution of GPCRs: Somatostatin/urotensin II receptors. J Mol Endocrinol. 52(3):T61-86.

**LGR**	Lamprey (a)	Fishes (Actinopterygii)				Fishes (Sarcopterygii)	Amphib-ian	Rep-tiles	Birds	Mammals	Comments
	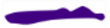	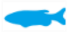	x2	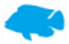	x2	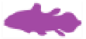	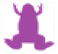	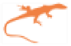	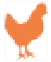	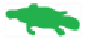 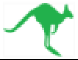 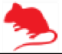 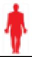	
RXFP1B		2	n	1†	n	1	1	2	3	2	Fishes annotated as rxfp2l. Good *et al.,* 2012
LHCGRb		1	n	9†	n						Maugars and Dufour, 2015

(a) at the root/near the root of a group/branch/cluster of subtypes, (†) spotted gar orthologue, (‡) local duplication

Good, S., Yegorov, S., Martijn, J., Franck, J., and Bogerd, J. (2012) New insights into ligand-receptor pairing and coevolution of relaxin family peptides and their receptors in teleosts. International Journal of Evolutionary Biology. 2012:310278.

Maugars, G., Dufour, S. (2015) Demonstration of the Coexistence of Duplicated LH Receptors in Teleosts, and Their Origin in Ancestral Actinopterygians. PLoS One. 10(8):e0135184.

**MRG**	Lamprey (a)	Fishes (Actinopterygii)	Fishes (Sarcopterygii)	Amphib-ian	Rep-tiles	Birds	Mammals	Comments
	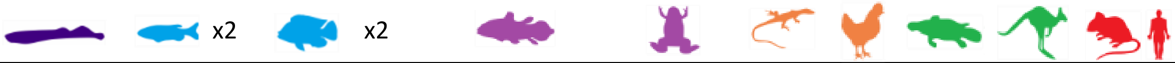	x2 x2						
MAS2					34‡	14‡		May be orthologous to mammalian MRGPR
MAS3					4‡			May be orthologous to MAS1
MAS4				5‡				May be orthologous to MAS1<br>Bader *et al.* 2014

(a) at the root/near the root of a group/branch/cluster of subtypes, (†) spotted gar orthologue, (‡) local duplication

Bader, M., Alenina, N., Andrade-Navarro, M.A., Santos, R.A. (2014) MAS and its related G protein-coupled receptors, Mrgprs. Pharmacol Rev. 66(4):1080-105.

**PURIN**	Lamprey (a)	Fishes (Actinopterygii)				Fishes (Sarcopterygii)	Amphibian	Reptiles	Birds	Mammals			Comments
	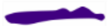	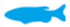	x2	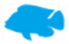	x2	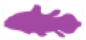	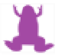	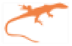	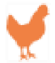	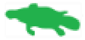	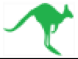	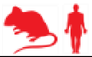	
GPR55B		2	n	1†	n	2‡			5	1			
CYSLTR3	3	3	n	6†	n	1							May include two subtypes.<br>Some species annotated as cysltr3.
GPR17B		2	n	7†	n								Annotated as si:dkey-96n2.3.
GPR183B		2	n	9†	n	2‡	1	1	4	1	1	3	Fishes annotated as si:ch211-184m13.4
P2RY10B											2	25	Mammalian-specific duplication<br>Some species annotated as A630033H20Rik.
P2RY1B		2	n	22†‡	n	1	3‡	2	5				Fishes annotated as si:dkey-78k11.9.
P2RY6B				4†	n	2‡		1				21	
GPR65B				5	n	1							
GPR132B		2	n	9†	n	1							Annotated GPR184.
GPR141B		2	n	9†	n								Most of the species annotated as si:dkey-94e7.1.
GPR141C						1		1	3		2	13	
GPR34b		2	n	1†	n		1	2	3				Fishes annotated as gpr34l. Schöneberg *et al.*, 2007, Schulz *et al.*, 2003
QRFPR2	1	2	n	1†	n	1							See Larhammar *et al.*, 2014, Ukena *et al.*, 2014
QRFPR3		3‡	n	10†‡	n	1	1	1					See Larhammar *et al.*, 2014, Ukena *et al.*, 2014

GPR176B		2	n	8	n		1					GPR176 sequences are not included in the guide trees.
HCAR4		2	n	9†	n	1						
FFAR2B		9‡	y	24†‡	y	1	1	1				Complex, may include several subtypes. Some of the fishes annotated as si:dkey211g8.6, si:ch211-231m23.4, and si:ch73-90p23.1
P2RY8B		1	n	1†	n	1	1	2				
F2RL4							1		5			
F2RB		7‡	n	11‡	n							
GPR196		2	n	2†‡	n	2‡						Updated in Ensembl.R92. Probably divided in two subtypes.
GPR195		2	n	3†	n	1		1				
GPR210		2	n	5†	n	1						
GPR213	2	3	y	16†	y	1	1					Fishes annotated as si:dkey-6n21.13.
GPR197	2						2‡					
GPR198		4	y	16†	n	2‡		1		2		Likely composed of two subtypes A and B Fishes annotated as si:dkey-165a24.9.
GPR199				1†	n	2‡		1	1	1	1	
GPR200		1	n	12†‡	n	2‡	1	3‡	2			Probably three subtypes A, B, C. Annotated as zmp:0000001084.

GPR212	6‡	n	35†‡	n					Zebrafishes annotated as si:dkey83h2.2, si:ch73-309g22.1, CR626884.1.
GPR202A	4	y	4†	n			3	1	
GPR202B			1†	n	2‡	1			

(a) at the root/near the root of a group/branch/cluster of subtypes, (†) spotted gar orthologue, (‡) local duplication

Larhammar, D., Xu, B., and Bergqvist, C.A. (2014) Unexpected multiplicity of QRFP receptors in early vertebrate evolution. Frontiers in Neuroendocrine Science. 8:337.

Schulz, A. and Schöneberg, T. (2003) The Structural Evolution of a P2Y-like G-protein-coupled Receptor. The Journal of biological chemistry. 278(37):35531-35541.

Schöneberg, T., Hermsdorf, T., Engemaier, E., Engel, K., Liebscher, I., Thor, D., Zierau, K., Römpler, H., Schulz, A. (2007) Structural and functional evolution of the P2Y(12)-like receptor group. Purinergic Signal. 3(4):255-68.

Ukena, K., Osugi, T., Leprince, J., Vaudry, H., and Tsutsui, K. (2014) Molecular evolution of GPCRs: 26Rfa/GPR103. Journal of molecular endocrinology. 52(3):T119-31.

OTHERS	Lamprey (a)	Fishes (Actinopterygii)				Fishes (Sarcopterygii)	Amphib-ian	Rep-tiles	Birds	Mammals	Comments
			x2		x2						
GPR22B		2	y	17†	y	1	1	1		1	May include several subtypes
GPER1B				7	n						Pinto *et al.*, 2017
GPR151B		2	n	8	n	1	1				Annotated as zmp:0000000801.
GPR61B						1	1				Some species annotated as si:dkeyp-111e5.4, si:ch211-213o11.11.
GPR20B		2	n	6	n						Updated in Ensembl.R92.
GPR20C		1	n	15†	n						Updated in Ensembl.R92.
GPR148B		2	n	2†‡		1		1	4	1	Updated in Ensembl.R92.
GPR203		1	n	8†‡	n	3‡					Might be a group of subtypes, three spotted gar.
GPR188		2	n	9†	n	1	1	1			Some species annotated as zgc:162592, might be a subtype for GPR21/52.

(a) at the root/near the root of a group/branch/cluster of subtypes, (†) spotted gar orthologue, (‡) local duplication

Pinto, P.I.S., Andrade, A.R., Estêvão, M.D., Alvarado, M.V., Felip, A., Power, D.M. (2017) Duplicated membrane estrogen receptors in the European sea bass (Dicentrarchus labrax): phylogeny, expression and regulation throughout the reproductive cycle. J Steroid Biochem Mol Biol. pii: S0960-0760(17)30404-1.
